# Supplementary material for: PR interval prolongation and 1-year mortality among emergency department patients: a multicentre transnational cohort study
Source: BMJ Open. 2021 Dec 14;11(12):e054238. doi: 10.1136/bmjopen-2021-054238 (PMC8672022; doi:10.1136/bmjopen-2021-054238)
Supplement: Supplementary data [file bmjopen-2021-054238supp001.pdf]

Supplementary Appendix

PR interval prolongation and one-year mortality in the emergency department: a multicenter transnational cohort study

Supplementary materials and methods

Appendix A – Data sources

ECGs were extracted from MUSE® Cardiology Information System (GE Healthcare, Wauwatosa, WI, USA) or Megacare Electrocardiographic (ECG) Management System (Simens-Elema, Stockholm, Sweden). Information regarding time of arrival to the ED, triage, course of admission, length of stay, and time of discharge were extracted from the logistic systems in the ED at the Region of Southern Denmark [1] and Region of Skåne.

A unique ten-digit personal civil registration number given in Denmark and Sweden allows individual cross-linkage between databases. Data regarding birth, emigration, and vital status was extracted from the Danish Civil Registration System [2] and the Swedish Population Registry [3].

Data concerning patient comorbidities were retrieved from the Danish National Patient Registry [4] and the Skåne Healthcare Register [5] which contains diagnoses from both primary and secondary healthcare sectors. Data regarding prescriptions came from the Danish National Prescription Registry [4] and the Swedish National Pharmacy Register [6].

Appendix B – ECG measurements

Danish ECGs were recorded and digitally stored in MUSE® Cardiology Information System (GE Healthcare, Wauwatosa, WI, USA) and processed by the Marquette 12SL algorithm[7]. Swedish ECG's were stored by either Marquette or Philips Diagnostic ECG (Philips) and analyzed by the DXL algorithm [8]. ECGs were recorded by pre-hospital medical staff or at either one of the two clinics and all patients who had an ECG recorded within +/- 4 hours of admission to the ED were included. In the case of multiple ECGs recorded on the same individual, the first ECG of acceptable quality was used in the study. Individuals presenting with the following ECGs, obtained by the 12SL algorithm, were excluded from the study: pacemaker or Implantable Cardioverter-Defibrillator (ICD) who had not been excluded by previous criteria (n = 5,448), those with atrial fibrillation and atrial flutter (n = 11,360), and missing PR intervals (n = 1,160). Missing PR interval on the ECG was due to bad quality ECG or nodal rhythm on ECG. Pacemaker or ICD were defined as previous history of pacemaker or ICD implantation (Z95.0) and/or pacemaker rhythm recorded on the ECG.

Furthermore, patients presenting with a PR interval <120 ms (n = 4,162) were excluded from the analysis in order to evaluate PR interval prolongation compared to a normal PR interval of 120-200 ms. The Marquette 12SL algorithm calculates a median PR interval from the 10-second tracing ECG starting at the lead showing the earliest depolarization of the atria to the earliest depolarization of the ventricles in any lead [7]

The ability of the 12SL algorithm to determine PR interval has been evaluated by GE in the Physician's Guide [7] and by Nielsen et al[9], who found a mean difference between manual and 12SL measured PR interval of -0,21 ms (95% CI, -1.17 ms to 0.76 ms) with limits of agreement (±2 standard deviations) ranging from -14.1 ms to 13.7 ms. The ability of the Marquette 12SL algorithm to determine the presence of atrial fibrillation has been validated by Hansen et al [10].

The mean PR interval measured in the cohort were 161.0 ms (SD: 29,35). The GE Marquette 12SL measured a mean of 160,6 ms (SD: 29.27) and the Philips DXL a mean of 161,54 ms (SD: 29,45). Figure B1 shows a visual presentation of the PR intervals measured by the two different algorithms. As mention GE Marquette 12SL has been validated, and the above show a strong correlation between the two algorithms.

Figure B:

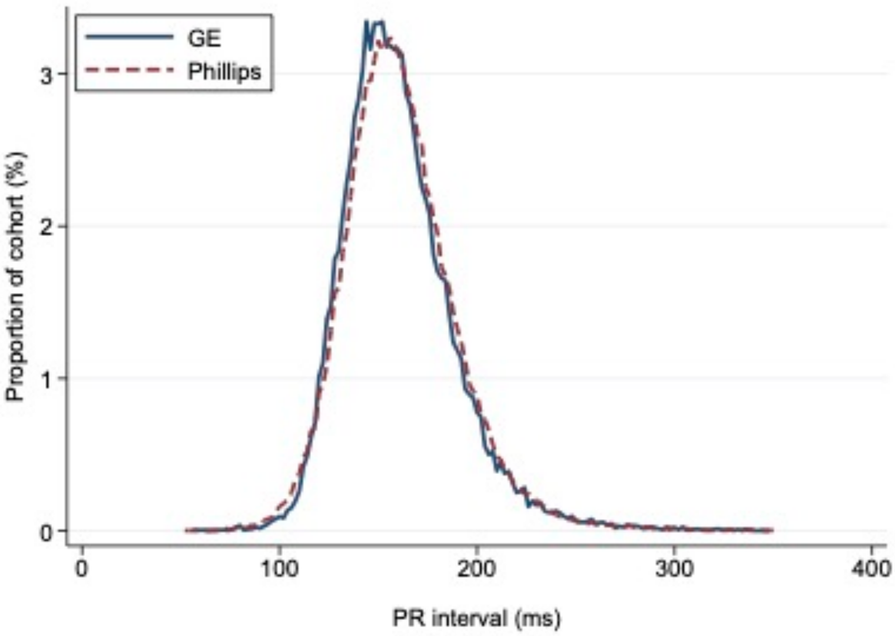

Distribution of the PR intervals measured by GE Marquette 12 SL (blue) and Philips DXL (red)

Appendix C – Codes and definitions

C.1: Charlson Comorbidity Index*

Condition	Assigned weight	ICD-10 codes
**Peripheral vascular disease**	1	I70.x, I71x, I73.1, I73.8, I73.9, I77.1, I79.0, I79.2, K55.1, K55.8, K55.9, Z95.8, Z95.9
**Cerebrovascular disease**	1	G45.x, G46.x, H34.0, I60.x-I69.x
**Dementia**	1	F00.x-F03.x, F05.1, G30.x, G31.1
**Chronic pulmonary disease**	1	I27.8, I27.9, J40.x-J47.x, J60.x-J67.x, J68.4, J70.1, J70.3
**Rheumatic disease**	1	M05.x, M06.x, M31.5, M32.x-M34.x, M35.1, M35.3, M36.0
**Peptic ulcer disease**	1	K25.x-K28.x
**Mild liver disease**	1	B18.x, K70.0-K70.3, K70.9, K71.3-K71.5, K71.7, K73.x, K74.x, K76.0, K76.2-K76.4, K76.8, K76.9, Z94.4
**Diabetes without chronic complications**	1	E10.0, E10.1, E10.6, E10.8, E10.9, E11.0, E11.1, E11.6, E11.8, E11.9, E12.0, E12.1, E12.6, E12.8, E12.9, E13.0, E13.1, E13.6, E13.8, E13.9, E14.0, E14.1, E14.6, E14.8, E14.9
**Hemiplegia or paraplegia**	2	G04.1, G11.4, G80.1, G80.2, G81.x, G82.x, G83.0-G83.4, G83.9
**Renal disease**	2	I12.0, I13.1, N03.2-N03.7, N05.2- N05.7, N18.x, N19.x, N25.0, Z49.0-Z49.2, Z94.0, Z99.2
**Diabetes with chronic complications**	2	E10.2-E10.5, E10.7, E11.2-E11.5, E11.7, E12.2-E12.5, E12.7, E13.2- E13.5, E13.7, E14.2-E14.5, E14.7
**Cancer**	2	C00.x-C26.x, C30.x-C34.x, C37.x- C41.x, C43.x, C45.x-C58.x, C60.x-C76.x, C81.x-C85.x, C88.x, C90.x-C97.x
**Metastatic cancer**	3	C77.x-C80.x
**Moderate or severe liver disease**	3	I85.0, I85.9, I86.4, I98.2, K70.4, K71.1, K72.1, K72.9, K76.5, K76.6, K76.7
**AIDS/HIV****	6	B20.x-B22.x, B24.x

**Diagnostic codes for myocardial infarction and heart failure are not included in the index but are included separately in this study as covariates.*
*** AIDS = acquired immunodeficiency syndrome, HIV = human immunodeficiency virus*

C.2: Myocardial infarction and congestive heart failure*

**Myocardial infarction**	I21.x, I22.x, I25.2
**Congestive heart failure**	I09.9, I11.0, I13.0, I13.2, I25.5, I42.0, I42.5-I42.9, I43.x, I50.x, P29.0

** Defined by Charlson Comorbidity Index but included separately in the study as covariates*

C.3: List of drugs associated with PR interval prolongation*

**Beta blocking agents**	C07*
**Calcium channel blockers**	C08*
**Digitalis glycosides**	C01AA*
**Adenosine**	C01EB10

**Usage of drugs are included dichotomously in the analysis defined as a redeemed prescription within 90 days of admission to the emergency department*

Appendix D – Supplementary results

Propensityscore matched cohort only adjusting for age and sex.

Tabel D.1,

	n	Events	HR (CI 95%)
Propensity score matched cohort*			
One-year all-cause mortality			
Normal PR 120-200 ms	24,996	3,248	1.0 (ref)
Prolonged PR >200 ms	8,321	893	0.96 (0.89-1.03)

Table D.2, sensitivity analysis

	Study cohort				Matched cohort	
	All	Prolonged PR >200 ms	Normal PR 120-180 ms	LowPR <120 ms	Prolonged PR >200 ms	Normal PR 120-180 ms
All	(n=92,089)	(n=9,397)	(n=78,530)	(n=4,162)	(n=7,016)	(n=21,048)
Male sex	43,648 (47.4%)	5,558 (59.1%)	36,521 (46.5%)	1,569 (37.7%)	3,621 (51.6%)	10,844 (51.5%)
Age, median (IQR)	60 (42-73)	75 (63-84)	58 (41-71)	52 (34-69)	70 (58-78)	69 (58-78)
18-50	33,693 (36.6%)	1,157 (12.3%)	30,537 (38.9%)	1,999 (48.0%)	1,157 (16.5%)	3,494 (16.6%)
51-69	29,430 (32.0%)	2,373 (25.3%)	25,886 (33.0%)	1,171 (28.1%)	2,349 (33.5%)	7,077 (33.6%)
70+	28,966 (31.5%)	5,867 (62.4%)	22,107 (28.2%)	992 (23.8%)	3,510 (50.0%)	10,477 (49.8%)
Charlson						
0	64,843 (70.4%)	5,785 (61.6%)	56,140 (71.5%)	2,918 (70.1%)	4,540 (64.7%)	13,584 (64.5%)
1	11,998 (13.0%)	1,309 (13.9%)	10,114 (12.9%)	575 (13.8%)	993 (14.2%)	2,983 (14.2%)
2	9,495 (10.3%)	1,494 (15.9%)	7,622 (9.7%)	379 (9.1%)	911 (13.0%)	2,804 (13.3%)
3+	5,753 (6.2%)	809 (8.6%)	4,654 (5.9%)	290 (7.0%)	572 (8.2%)	1,677 (8.0%)
Other diagnoses						
Heart failure	3,651 (4.0%)	950 (10.1%)	2,534 (3.2%)	167 (4.0%)	276 (3.9%)	839 (4.0%)
MI	4,019 (4.4%)	913 (9.7%)	2,967 (3.8%)	139 (3.3%)	351 (5.0%)	1,120 (5.3%)
Use of medication						
QT-prolong drugs	15,281 (16.6%)	2,886 (30.7%)	11,912 (15.2%)	483 (11.6%)	1,638 (23.3%)	4,961 (23.6%)
Center						
Odense	11,850 (12.9%)	1,146 (12.2%)	10,162 (12.9%)	542 (13.0%)	886 (12.6%)	2,658 (12.6%)
South West Jutland	8,559 (9.3%)	932 (9.9%)	7,337 (9.3%)	290 (7.0%)	693 (9.9%)	2,079 (9.9%)
Skåne	37,541 (40.8%)	3,960 (42.1%)	31,682 (40.3%)	1,899 (45.6%)	2,801 (39.9%)	8,403 (39.9%)
Helsingborg	34,139 (37.1%)	3,359 (35.7%)	29,349 (37.4%)	1,431 (34.4%)	2,636 (37.6%)	7,908 (37.6%)
ECG HR, median (IQR)	77 (66-90)	71 (62-83)	77 (67-90)	85 (70-103)	71 (61-83)	78 (67-91)

Supplementary results showing baseline characteristics in patients with normal PR interval (120-180 ms) and PR interval prolongation (>200 ms).

Appendix E – Post hoc analysis

Cardiovascular events

	n	Events	HR (CI 95%)
Propensity score matched cohort			
One year risk of MI			
Normal PR 120-200 ms	22,862	1,354	1.0 (ref)
Prolonged PR >200 ms	7,651	521	1.26 (1.07-1.49)
One year risk of CHF			
Normal PR 120-200 ms	23,084	1,238	1.0 (ref)
Prolonged PR >200 ms	7,724	456	1.16 (1.01-1.34)

Association between PR interval prolongation and myocardial infarction (MI) and congestive heart failure (CHF). MI and CHF are defined as listed under table C.2

References

1. Norgaard B, Mogensen CB, Teglbjaerg LS, et al. Diagnostic packages can be assigned accurately in emergency departments. A multi-centre cohort study. *Dan Med J* 2016;63(6) [published Online First: 2016/06/07]
2. Schmidt M, Pedersen L, Sorensen HT. The Danish Civil Registration System as a tool in epidemiology. *Eur J Epidemiol* 2014;29(8):541-9. doi: 10.1007/s10654-014-9930-3 [published Online First: 2014/06/27]
3. Ludvigsson JF, Otterblad-Olausson P, Pettersson BU, et al. The Swedish personal identity number: possibilities and pitfalls in healthcare and medical research. *Eur J Epidemiol* 2009;24(11):659-67. doi: 10.1007/s10654-009-9350-y [published Online First: 2009/06/09]
4. Schmidt M, Schmidt SA, Sandegaard JL, et al. The Danish National Patient Registry: a review of content, data quality, and research potential. *Clin Epidemiol* 2015;7:449-90. doi: 10.2147/clep.s91125 [published Online First: 2015/11/26]
5. Löfvendahl S, Schelin MEC, Jöud A. The value of the Skåne Health-care Register: Prospectively collected individual-level data for population-based studies. *Scand J Public Health* 2020;48(1):56-63. doi: 10.1177/1403494819868042 [published Online First: 2019/12/10]
6. Astrand B, Hovstadius B, Antonov K, et al. The Swedish National Pharmacy Register. *Stud Health Technol Inform* 2007;129(Pt 1):345-9. [published Online First: 2007/10/04]
7. Marquette 12SL ECG Analysis Program: Physician’s Guide. Revision B 2056246-002. https://www.gehealthcare.com: GE Healthcare, 2015:428.
8. Philips DXL ECG Algorithm Physician’s Guide. 2 ed. Andover MA: Philips Medical Systems, 2009.
9. Nielsen JB, Pietersen A, Graff C, et al. Risk of atrial fibrillation as a function of the electrocardiographic PR interval: results from the Copenhagen ECG Study. *Heart Rhythm* 2013;10(9):1249-56. doi: 10.1016/j.hrthm.2013.04.012 [published Online First: 2013/04/24]
10. Hansen TG, AP MS, Brandes A, et al. New-onset atrial fibrillation among patients with infection in the emergency department: A multicentre cohort study of one-year stroke risk. *Am J Med* 2019 doi: 10.1016/j.amjmed.2019.06.048 [published Online First: 2019/08/14]
