# Supplementary material for: PR interval prolongation and 1-year mortality among emergency department patients: a multicentre transnational cohort study
Source: BMJ Open. 2021 Dec 14;11(12):e054238. doi: 10.1136/bmjopen-2021-054238 (PMC8672022; doi:10.1136/bmjopen-2021-054238)
Supplement: Author's manuscript [file bmjopen-2021-054238.draft_revisions.pdf]

BMJ Open

BMJ Open is committed to open peer review. As part of this commitment we make the peer review history of every article we publish publicly available.

When an article is published we post the peer reviewers' comments and the authors' responses online. We also post the versions of the paper that were used during peer review. These are the versions that the peer review comments apply to.

The versions of the paper that follow are the versions that were submitted during the peer review process. They are not the versions of record or the final published versions. They should not be cited or distributed as the published version of this manuscript.

BMJ Open is an open access journal and the full, final, typeset and author-corrected version of record of the manuscript is available on our site with no access controls, subscription charges or pay-per-view fees (http://bmjopen.bmj.com).

If you have any questions on BMJ Open's open peer review process please email info.bmjopen@bmj.com

BMJ Open

PR interval prolongation and one-year mortality among emergency department patients: a multicenter transnational cohort study

Journal:	*BMJ Open*
Manuscript ID	bmjopen-2021-054238
Article Type:	Original research
Date Submitted by the Author:	10-Jun-2021

Complete List of Authors:	Pedersen, Rune; Odense University Hospital, 1. Department of Emergency Medicine<br>Larsen, Tobias; Odense University Hospital, Department of Emergency Medicine<br>Jensen, Helene; University of Southern Denmark, Clinical Pharmacology, Pharmacy, and Environmental Medicine, Department of Public Health,<br>Brabrand, Mikkel; Southwest Jutland Hospital Esbjerg, Department of Emergency Medicine<br>Lundager Forberg, Jakob; Helsingborgs lasarett, Department of Emergency Medicine and Prehospital Care<br>Ekelund, U; Lunds Universitet, Department of Emergency Medicine<br>Pottegard, Anton; University of Southern Denmark, Clinical Pharmacology, Pharmacy, and Environmental Medicine, Department of Public Health<br>Lassen, Annmarie; Odense University Hospital, Department of Emergency Medicine

Keywords:	ACCIDENT & EMERGENCY MEDICINE, Cardiac Epidemiology < CARDIOLOGY, EPIDEMIOLOGY

SCHOLARONE™
Manuscripts

I, the Submitting Author has the right to grant and does grant on behalf of all authors of the Work (as defined in the below author licence), an exclusive licence and/or a non-exclusive licence for contributions from authors who are: i) UK Crown employees; ii) where BMJ has agreed a CC-BY licence shall apply, and/or iii) in accordance with the terms applicable for US Federal Government officers or employees acting as part of their official duties; on a worldwide, perpetual, irrevocable, royalty-free basis to BMJ Publishing Group Ltd ("BMJ") its licensees and where the relevant Journal is co-owned by BMJ to the co-owners of the Journal, to publish the Work in this journal and any other BMJ products and to exploit all rights, as set out in our licence.

The Submitting Author accepts and understands that any supply made under these terms is made by BMJ to the Submitting Author unless you are acting as an employee on behalf of your employer or a postgraduate student of an affiliated institution which is paying any applicable article publishing charge ("APC") for Open Access articles. Where the Submitting Author wishes to make the Work available on an Open Access basis (and intends to pay the relevant APC), the terms of reuse of such Open Access shall be governed by a Creative Commons licence – details of these licences and which Creative Commons licence will apply to this Work are set out in our licence referred to above.

Other than as permitted in any relevant BMJ Author's Self Archiving Policies, I confirm this Work has not been accepted for publication elsewhere, is not being considered for publication elsewhere and does not duplicate material already published. I confirm all authors consent to publication of this Work and authorise the granting of this licence.

For peer review only

PR interval prolongation and one-year mortality among emergency department patients: a multicenter transnational cohort study

Rune Vad Pedersen [CO, 1]
Tobias Malte Larsen [CO, 1]
Helene Kildegaard Jensen[2], Mikkel Brabrand[1,3], Jakob Lundager Forberg[4], Ulf Ekelund[5], Anton Pottegård[2], Annmarie Touborg Lassen[1]

Author affiliations:

1. Department of Emergency Medicine, Odense University Hospital, Odense, Denmark
2. Clinical Pharmacology, Pharmacy, and Environmental Medicine, Department of Public Health, University of Southern Denmark, Odense, Denmark
3. Department of Emergency Medicine, Hospital of South West Jutland, Esbjerg, Denmark
4. Department of Emergency Medicine, Helsingborg Hospital, Helsingborg, Sweden
5. Department of Emergency Medicine, Skåne University Hospital, Lund, Sweden

Corresponding author

Rune Vad Pedersen

Hjallesevej 54 2.sal, 5230 Odense M

rune@vadpedersen.dk

Tel. 0045 30296343

Word count: 2011

Abstract word count: 225

For peer review only

Abstract

Objectives

Emerging evidence supports that PR interval prolongation is associated with increased mortality. However, most previous studies have limited confounder control, and clinical impact in a population of acute ill patients is unknown. The aim of this study was to investigate whether one-year all-cause mortality was increased in patients presenting with PR interval prolongation in the emergency department (ED).

Methods

We conducted a register-based cohort study in two Swedish and two Danish EDs. We included all adult patients with an ECG performed at arrival to the Danish EDs during March 2013 to May 2014 and Swedish EDs during January 2010 to January 2011. Using propensity score matching, we analyzed hazard ratio (HR) for one-year all-cause mortality comparing patients with PR interval prolongation (>200 ms) and normal PR interval (120-200 ms).

Results

We included 106,113 patients. PR interval prolongation occurred in 8.9% (95% CI, 8.7-9.0); these patients were older and had more co-morbidity than those with a normal PR interval. The absolute one-year risk of death was 13% (95% CI, 12.3-13.7) for patients with PR interval prolongation and 7.9% (95% CI 7.7-8.0) for those without. After confounder adjustments by propensity score matching, PR interval prolongation showed no association with one-year mortality with a HR of 1.00 (95% CI, 0.93-1.08).

Conclusion

PR interval prolongation does not constitute an independent risk factor for one-year mortality in ED patients.

For peer review only

Strengths and limitations

- Large multicenter transnational study population
- Cross-linkage of several databases
- Comprehensive statistical analysis including a propensity score matched cohort
- Due to the nature of the study design causality cannot be assessed
- No stratification by cause of admission to the ED

Introduction

As a measure of conduction time from the onset of atrial depolarization to the beginning of ventricular depolarization, the PR interval reflects the propagation of electrical impulses from the sinus node to the ventricles. A delay in the propagation of electrical impulses resulting in PR interval prolongation (>200 ms) is clinically known as first-degree atrioventricular block or delay.[1] PR interval prolongation has clinically been considered a benign condition, but recent studies provide increasing evidence that PR interval prolongation in different populations is a predictor of future atrial fibrillation, implantation of a pacemaker or an implantable cardioverter-defibrillator, major cardiac events, and all-cause mortality.[2] The most broadly accepted pathogenetic explanation of a possible association between PR interval prolongation and mortality is that age-related myocardial fibrosis plays a role in delayed electric conduction and increased vulnerability to arrhythmia.[3]

The subject is still debated, and the clinical consequence of a possible increased risk is unclear. Most prior studies have been performed in healthy populations with 5-35 years of follow-up.[4-6] No previous studies have evaluated the prognostic significance of PR interval prolongation among acutely ill patients.

In this study, we hypothesized that PR interval prolongation is associated with an increased risk of one-year all-cause mortality in patients presenting to the emergency department (ED).

For peer review only - http://bmjopen.bmj.com/site/about/guidelines.xhtml

Materials and methods

Study design and setting

This was a register-based multi-centre cohort study based on adult ED patients in the two Danish hospitals Odense University Hospital (OUH) and Hospital of South West Jutland (SVS) and the two Swedish hospitals Skåne University Hospital (SUS) at Lund and Helsingborg Hospital (HH). The study was based on Danish data from 1st of March 2013 to 1st of May 2014 and Swedish data from 1st of January 2010 to 1st of January 2011. OUH covers a population of 270,000 people while SVS covers a population of 220,000 people. SUS Lund covers 270,000 people while the regional hospital HH covers 250,000.

In Denmark and Sweden, the health care system is tax-funded; hence, all residents have free access to healthcare, though with a small co-payment in Sweden.

Selection of patients

All adult patients (≥ 18 years), who had a 12-lead ECG recorded within 4 hours of arrival to the ED, were eligible for the study. In case of multiple recordings, the first ECG taken within this period was used. Patients with multiple contacts within the study period were included only at their first contact. Contacts with invalid Civil Personal Registration (CPR) number and patients aged <18 years were excluded. Patients with loss to follow-up due to emigration were censored. Furthermore, patients with a pacemaker, atrial fibrillation or flutter, and missing PR intervals were excluded (Figure 1).

Data sources

A unique CPR number is given to every resident in Denmark and Sweden and used in all hospital contacts, which allows individual cross-linkage between databases. ECGs were extracted from electronic central databases at the Region of Southern Denmark and Region of Skåne. Logistic information was extracted from the logistic systems at the EDs of the Region of Skåne and Region of Southern Denmark.[7] Patient comorbidities were retrieved from the Danish National Patient Registry[8] and the Skåne Healthcare Databases, data regarding prescriptions from the Danish National Prescription Registry[9] and the Swedish Pharmacy Registry,[10] and data regarding birth,

For peer review only - http://bmjopen.bmj.com/site/about/guidelines.xhtml

emigration, and vital status were obtained from the Danish Civil Registration System[11] and the Swedish Population Register.[12] Appendix A provides further details regarding data sources.

Definitions and ECG measurements

A normal PR interval was defined as 120-200 ms.[1] PR interval prolongation was defined as PR interval > 200 ms.[1] The PR interval was automatically calculated as a median value by the GE Marquette 12SL ECG Analysis Program or Philips DXL and stored in the MUSE Cardiology Information System or the Megacare Electrocardiographic (ECG) Management System (Simens-Elema, Stockholm, Sweden), respectively. The ability of the Marquette 12SL algorithm to determine PR interval has been evaluated by GE[13] and by Nielsen et al.[14] who found a mean difference of -0.21 ms (95% CI: -1.17 - 0.76 ms) compared to manual measurement. Appendix B provides further information on ECG measurements.

Comorbidity status was defined by the Charlson Comorbidity Index (CCI,[15 16]) with exclusion of myocardial infarction and congestive heart failure which were used as separate covariates in the propensity score matching. Appendix C.1 provides further information on CCI. Usage of PR interval prolonging drugs was defined as redeemed prescriptions of these drugs within the last 90 days (appendix C.3).

Statistics and primary analysis

Continuous data are presented as medians with interquartile ranges (IQR) and categorical data as proportions with 95% CIs based on a binomial distribution. The mortality of patients with PR interval prolongation was compared with patients having a normal PR interval.

Patients were followed from ED visit until death or 365 days, whichever came first. Survival was represented by a Kaplan-Meier survival curve. Furthermore, the crude relationship between PR interval and one-year all-cause mortality was illustrated using restricted cubic splines.[17] Unadjusted one-year all-cause mortality was presented as absolute risk and assessed using univariate Cox regression comparing patients with PR interval prolongation (>200 ms) and normal PR interval (120-200 ms). Propensity scores were estimated for each individual by use of logistic regression with a PR interval of > 200 ms as a binary outcome. The following covariates were included in the logistic regression model: sex, age, CCI, myocardial infarction, congestive heart failure, study centre, and PR interval prolonging drugs. We generated a propensity matched cohort by a 1:3 parallel, balanced, nearest neighbor matching with a caliper of 0.02, and estimated hazards ratios

For peer review only - http://bmjopen.bmj.com/site/about/guidelines.xhtml

for one-year mortality using Cox regression. Heart rate was adjusted for after propensity score matching by multivariate regression.

All statistical tests were 2-sided with p-values < 0.05 considered statistically significant. Stata version 16 (StataCorp, College Station, TX, USA) was used for analyses.

Sensitivity analyses

30 days mortality was calculated based on the propensity score matched cohort. As a sensitivity analysis, a second propensity score matched cohort was created after exclusion of patients with PR intervals between 180 and 199.

Ethics

The study was approved by Region of Southern Denmark (Journal nr. 19/16024) and the Danish Health Authority (No. 3-3013-1031). In accordance with Swedish law, the study was approved by the regional ethics review board at Lund and by Region Skåne (Dnr 2015/129).

Patient and public involvement

This was a study without direct patient contact.

For peer review only

For peer review only - http://bmjopen.bmj.com/site/about/guidelines.xhtml

Results

We identified 124,336 ED contacts within the study period. A total of 106,113 patients were included in the study. Reasons for exclusion are presented in Figure 1. Among patients included in the study, PR interval prolongation (>200 ms) occurred in 9,398 (9%) patients. Among those with PR interval prolongation, the average age was 75 years and 59% were male. Patients with PR interval prolongation were generally older, more likely to be male, had more comorbidities, and took more medications than those with a normal PR interval (Table 1).

In the propensity score matched cohort, 33,004 patients were included. A total of 8,251 patients with PR interval prolongation were matched with 24,753 patients with a normal PR interval (Table 1).

Prognosis

With 8,830 deaths in the cohort, one-year all-cause mortality was 8.3% (95% CI: 8.2 to 8.5). The cubic spline showed a U-shaped association between PR interval and mortality (Figure 2).

Among 9,398 patients with PR interval prolongation, 1,219 died, representing a 13% (95% CI,12.3-13.7) absolute one-year risk of death. Among 96,715 patients with normal PR interval, 7,611 died representing an 7.9 % (95% CI: 7.7 to 8.0) absolute risk of death. The unadjusted relative risk of death given PR interval prolongation was 1.65 (95% CI: 1.56 - 1.75; Figure 3).

In the heart rate adjusted propensity score matched cohort, PR interval prolongation had a HR of 1.00 (95% CI 0.93 - 1.08) for one-year mortality (Table 2) and a HR of 1.11 (95% CI 0.97 to 1.28) for 30-day all-cause mortality.

Sensitivity analyses

After exclusion of PR intervals ranging from 180-199 ms, patients with PR interval prolongation had a HR of 0.90 (95% CI: 0.82 - 0.99) for one-year mortality (Table 2). Appendix D provides supplementary baseline characteristics.

Discussion

With a focus on one-year all-cause mortality, the present study was designed to investigate the transferability of results from prior studies onto acutely ill patients. We found that PR interval prolongation did not constitute an independent risk factor for one-year all-cause mortality among

For peer review only

unselected ED patients. This result corresponds to the clinical practice where no interventions are needed with regards to asymptomatic patients with first-degree atrioventricular block.[1]

Our result differs from previous studies in healthy populations which suggest a possible association between prolonged PR interval and significant increases in atrial fibrillation, heart failure and mortality.[2] The diverging results may be due to important differences in study design and population. Most previous studies of prolonged PR interval provide information of 5-35 years mortality,[2 4 18] whereas this is the first study of PR interval prolongation and prognosis in an ED setting. The unique civil registration number allowed cross-linkage between different databases which resulted in very little loss to follow-up. Hazard ratios were calculated on the heart rate adjusted propensity score matched cohort, and this represents a thorough confounder control which contributes to the robustness of the statistical analysis. Few prior studies[18 19] have included this level of confounder control. We also conducted a sensitivity analysis using a "twilight" which excluded individuals with a PR interval of 180-199 ms and supported our main analysis. Our results thus indicate that the increased unadjusted relative risk is due to confounders and not PR interval prolongation. Inclusion of patients from four centers, two tertiary referral centers (OUH and SUS) and two regional hospitals (SVS and HH), strengthen the external validity. Further, a relatively large study population compared to prior studies[4 6 18 20] improves the precision of the results.

In our study we found an increased absolute risk of one-year mortality in patients with very short as well as prolonged PR interval (Figure 2). On an individual patient level, the unadjusted absolute risk of one-year death was increased by 65% given a PR interval prolongation. The correlation between a higher heart rate and shorter PR interval may explain the increased probability of death among patients with short PR interval. Furthermore, diseases with ventricular pre-excitation cause shortening of the PR interval and might also contribute, but pre-excitatory syndromes like Wolff-Parkinson-White are very rare, and are unlikely to contribute to the present observation.[21] In the main analysis heart rate was adjusted for after propensity score matching, and thereby did not influence the results.

The present study has some limitations. An observational study cannot investigate causality but only test and generate hypotheses. Though propensity score matching has some strengths, it does not balance unmeasured confounders. Of note, BMI, smoking status, blood pressure, ethnicity, and socioeconomic status were not possible to extract from the database.

For peer review only - http://bmjopen.bmj.com/site/about/guidelines.xhtml

Further, an ECG performed in the ED is just a snapshot, as the PR interval could have been different just hours earlier and it is therefore unknown whether PR interval prolongation regress, persists, or develops into more severe cardiac disease. Also, the PR interval varies with circadian rhythm.[22] Although this variation could result in misclassification, it is expected to distribute evenly between patients with prolonged and normal PR interval.

We did not analyze the hazards in different patient subgroups, e.g. based on chief complaint at the ED or comorbidities. In this context, it is reasonable to believe that PR interval prolongation may be a better predictor for adverse events in individuals presenting with syncope, presyncope, palpitations etc.[23] It is unclear whether these patients would benefit from closer monitoring, e.g. with an ECG every 2-5 years, as previously suggested.[2]

The population in Denmark and Sweden have a universal access to the healthcare system. Generalization of our results outside of Western Europe is therefore uncertain. Another limitation is that most of the patients in the present study are Caucasians. PR interval has been shown to be a more sensitive predictor of atrial fibrillation among African Americans.[24]

In conclusion, PR interval prolongation did not constitute an independent risk factor for one-year all-cause mortality among unselected ED patients.

For peer review only

Contributors

Co-First/Equal authorship: TML and RVP contributed equally to this work. TML, RVP, AP and AL designed the study, interpreted the results and TML and RVP drafted the paper. TML, RVP, AP and HJ analyzed the data. UE, ATL, MB, and JLF created the EXPECT database. All authors critically reviewed the paper, assisted with interpretation of the results and approved the final edition.

Conflicts of interest

The authors declare no conflicts of interest.

Data sharing

Due to the danish law on personal data, we are not allowed to share data in a public dataset. All data relevant to the study are included in the article or uploadet as supplementary information.

Funding

The work was supported by Odense University Hospital and the Swedish heart-Lung Foundation. This study was also part of the AIR Lund (Artificially Intelligent use of Resisters at Lund University) research environment and received funding from the Swedish Research Council (VR; grant no. 2019-00198).

For peer review only

References

1. Kusumoto FM, Schoenfeld MH, Barrett C, et al. 2018 ACC/AHA/HRS Guideline on the Evaluation and Management of Patients With Bradycardia and Cardiac Conduction Delay. *Circulation* 2018:Cir0000000000000628. doi: 10.1161/cir.0000000000000628 [published Online First: 2018/12/28]
2. Kwok CS, Rashid M, Beynon R, et al. Prolonged PR interval, first-degree heart block and adverse cardiovascular outcomes: a systematic review and meta-analysis. *Heart (British Cardiac Society)* 2016;102(9):672-80. doi: 10.1136/heartjnl-2015-308956 [published Online First: 2016/02/18]
3. de Jong S, van Veen TA, van Rijen HV, et al. Fibrosis and cardiac arrhythmias. *J Cardiovasc Pharmacol* 2011;57(6):630-8. doi: 10.1097/FJC.0b013e318207a35f [published Online First: 2010/12/15]
4. Aro AL, Anttonen O, Kerola T, et al. Prognostic significance of prolonged PR interval in the general population. *European heart journal* 2014;35(2):123-9. doi: 10.1093/eurheartj/eht176 [published Online First: 2013/05/17]
5. Chan YH, Hai JJ, Lau KK, et al. PR interval prolongation in coronary patients or risk equivalent: excess risk of ischemic stroke and vascular pathophysiological insights. *BMC Cardiovasc Disord* 2017;17(1):233. doi: 10.1186/s12872-017-0667-2 [published Online First: 2017/08/25]
6. Magnani JW, Wang N, Nelson KP, et al. Electrocardiographic PR interval and adverse outcomes in older adults: the Health, Aging, and Body Composition study. *Circ Arrhythm Electrophysiol* 2013;6(1):84-90. doi: 10.1161/circep.112.975342 [published Online First: 2012/12/18]
7. Norgaard B, Mogensen CB, Teglbjaerg LS, et al. Diagnostic packages can be assigned accurately in emergency departments. A multi-centre cohort study. *Dan Med J* 2016;63(6) [published Online First: 2016/06/07]
8. Schmidt M, Schmidt SA, Sandegaard JL, et al. The Danish National Patient Registry: a review of content, data quality, and research potential. *Clin Epidemiol* 2015;7:449-90. doi: 10.2147/clep.s91125 [published Online First: 2015/11/26]
9. Pottegård A, Hallas J, Sørensen HT, et al. Data Resource Profile: The Danish National Prescription Registry. *International Journal of Epidemiology* 2016;46(3):798-98f. doi: 10.1093/ije/dyw213
10. Astrand B, Hovstadius B, Antonov K, et al. The Swedish National Pharmacy Register. *Stud Health Technol Inform* 2007;129(Pt 1):345-9. [published Online First: 2007/10/04]
11. Schmidt M, Pedersen L, Sorensen HT. The Danish Civil Registration System as a tool in epidemiology. *Eur J Epidemiol* 2014;29(8):541-9. doi: 10.1007/s10654-014-9930-3 [published Online First: 2014/06/27]
12. Ludvigsson JF, Otterblad-Olausson P, Pettersson BU, et al. The Swedish personal identity number: possibilities and pitfalls in healthcare and medical research. *Eur J Epidemiol* 2009;24(11):659-67. doi: 10.1007/s10654-009-9350-y [published Online First: 2009/06/09]
13. Marquette 12SL ECG Analysis Program: Physician's Guide. Revision B 2056246-002. https://www.gehealthcare.com: GE Healthcare, 2015:428.

For peer review only

14. Nielsen JB, Pietersen A, Graff C, et al. Risk of atrial fibrillation as a function of the electrocardiographic PR interval: results from the Copenhagen ECG Study. *Heart Rhythm* 2013;10(9):1249-56. doi: 10.1016/j.hrthm.2013.04.012 [published Online First: 2013/04/24]
15. Quan H, Sundararajan V, Halfon P, et al. Coding algorithms for defining comorbidities in ICD-9-CM and ICD-10 administrative data. *Med Care* 2005;43(11):1130-9. doi: 10.1097/01.mlr.0000182534.19832.83 [published Online First: 2005/10/15]
16. Charlson ME, Pompei P, Ales KL, et al. A new method of classifying prognostic comorbidity in longitudinal studies: development and validation. *J Chronic Dis* 1987;40(5):373-83. doi: 10.1016/0021-9681(87)90171-8 [published Online First: 1987/01/01]
17. Durrleman S, Simon R. Flexible regression models with cubic splines. *Stat Med* 1989;8(5):551-61. doi: 10.1002/sim.4780080504 [published Online First: 1989/05/01]
18. Cheng S, Keyes MJ, Larson MG, et al. Long-term outcomes in individuals with prolonged PR interval or first-degree atrioventricular block. *Jama* 2009;301(24):2571-7. doi: 10.1001/jama.2009.888 [published Online First: 2009/06/25]
19. Aro AL, Anttonen O, Kerola T, et al. Prognostic significance of prolonged PR interval in the general population. *European heart journal* 2013;35(2):123-29. doi: 10.1093/eurheartj/eht176
20. Hisamatsu T, Miura K, Fujiyoshi A, et al. Long-term outcomes associated with prolonged PR interval in the general Japanese population. *Int J Cardiol* 2015;184:291-3. doi: 10.1016/j.ijcard.2015.02.028 [published Online First: 2015/03/04]
21. Mark DG, Brady WJ, Pines JM. Preexcitation syndromes: diagnostic consideration in the ED. *Am J Emerg Med* 2009;27(7):878-88. doi: 10.1016/j.ajem.2008.06.013 [published Online First: 2009/08/18]
22. Dilaveris PE, Farbom P, Batchvarov V, et al. Circadian behavior of P-wave duration, P-wave area, and PR interval in healthy subjects. *Ann Noninvasive Electrocardiol* 2001;6(2):92-7. [published Online First: 2001/05/03]
23. Lewalter T, Purerfellner H, Ungar A, et al. "First-degree AV block-a benign entity?" Insertable cardiac monitor in patients with 1st-degree AV block reveals presence or progression to higher grade block or bradycardia requiring pacemaker implant. *J Interv Card Electrophysiol* 2018;52(3):303-06. doi: 10.1007/s10840-018-0439-7 [published Online First: 2018/08/15]
24. Shulman E, Aagaard P, Kargoli F, et al. Validation of PR interval length as a criterion for development of atrial fibrillation in non-Hispanic whites, African Americans and Hispanics. *J Electrocardiol* 2015;48(4):703-9. doi: 10.1016/j.jelectrocard.2015.04.015 [published Online First: 2015/05/31]

For peer review only

For peer review only - http://bmjopen.bmj.com/site/about/guidelines.xhtml

Tables and figures:

Table 1: Baseline characteristics

	Study cohort				Matched cohort	
	All	Prolonged PR >200 ms	Normal PR 120-200 ms	Short PR <120 ms	Prolonged PR >200 ms	Normal PR 120-200 ms
All	(n=106,113)	(n=9,398)	(n=92,555)	(n=4,160)	(n=8,251)	(n=24,753)
Male sex	51,156 (48.2%)	5,554 (59.1%)	44,029 (47.6%)	1,573 (37.8%)	4,581 (55.5%)	13,709 (55.4%)
Age, median (IQR)	61 (43-74)	75 (63-84)	59 (43-73)	52 (34-69)	72 (61-81)	72 (60-81)
18-50	36,752 (34.6%)	1,160 (12.3%)	33,595 (36.3%)	1,997 (48.0%)	1,160 (14.1%)	3,493 (14.1%)
51-69	34,169 (32.2%)	2,372 (25.2%)	30,627 (33.1%)	1,170 (28.1%)	2,369 (28.7%)	7,257 (29.3%)
70+	35,192 (33.2%)	5,866 (62.4%)	28,333 (30.6%)	993 (23.9%)	4,722 (57.2%)	14,003 (56.6%)
Charlson						
0	74,386 (70.1%)	5,787 (61.6%)	65,685 (71.0%)	2,914 (70.0%)	5,173 (62.7%)	15,633 (63.2%)
1	13,752 (13.0%)	1,308 (13.9%)	11,871 (12.8%)	573 (13.8%)	1,162 (14.1%)	3,447 (13.9%)
2	11,240 (10.6%)	1,496 (15.9%)	9,363 (10.1%)	381 (9.2%)	1,200 (14.5%)	3,598 (14.5%)
3+	6,735 (6.3%)	807 (8.6%)	5,636 (6.1%)	292 (7.0%)	716 (8.7%)	2,075 (8.4%)
Other diagnoses						
Heart failure	4,470 (4.2%)	949 (10.1%)	3,352 (3.6%)	169 (4.1%)	520 (6.3%)	1,665 (6.7%)
MI	4,931 (4.6%)	915 (9.7%)	3,877 (4.2%)	139 (3.3%)	594 (7.2%)	1,885 (7.6%)
Use of medication						
QT-prolong drugs	18,558 (17.5%)	2,887 (30.7%)	15,188 (16.4%)	483 (11.6%)	2,198 (26.6%)	6,736 (27.2%)
Center						
Odense	13,570 (12.8%)	1,146 (12.2%)	11,882 (12.8%)	542 (13.0%)	980 (11.9%)	2,940 (11.9%)
South West Jutland	9,907 (9.3%)	932 (9.9%)	8,685 (9.4%)	290 (7.0%)	815 (9.9%)	2,445 (9.9%)
Skåne	43,615 (41.1%)	3,959 (42.1%)	37,761 (40.8%)	1,895 (45.6%)	3,382 (41.0%)	10,146 (41.0%)
Helsingborg	39,021 (36.8%)	3,361 (35.8%)	34,227 (37.0%)	1,433 (34.4%)	3,074 (37.3%)	9,222 (37.3%)
ECG HR, median (IQR)	76 (66-89)	71 (62-83)	76 (66-90)	85 (70-103)	71 (62-83)	77 (66-90)

Table 1 Baseline characteristics of the study cohort and the propensity score matched cohort.

**PR = PR interval*

For peer review only

Table 2:

	n	Events	HR (CI 95%)
Propensity score matched cohort*			
One-year all-cause mortality			
Normal PR 120-200 ms	24,753	3,129	1.0 (ref)
Prolonged PR >200 ms	8,251	895	1.00 (0.93-1.08)
30 days all-cause mortality			
Normal PR 120-200 ms	24,753	1,042	1.0 (ref)
Prolonged PR >200 ms	8,251	308	1.11 (0.97-1.28)
Sensitivity analysis**			
One-year all-cause mortality			
Normal PR 120-180 ms	21,063	2,367	1.0 (ref)
Severe PR >220 ms	7,021	606	0.90 (0.82-0.99)

*Table 2 Risk assessment in the study population. *Heart rate adjusted propensity score matched cohort. **Sensitivity analysis on heart rate adjusted propensity score matched cohort excluding PR intervals between 180-199 ms (supplementary D)*

For peer review only

For peer review only - http://bmjopen.bmj.com/site/about/guidelines.xhtml

Figure 1: Flowchart

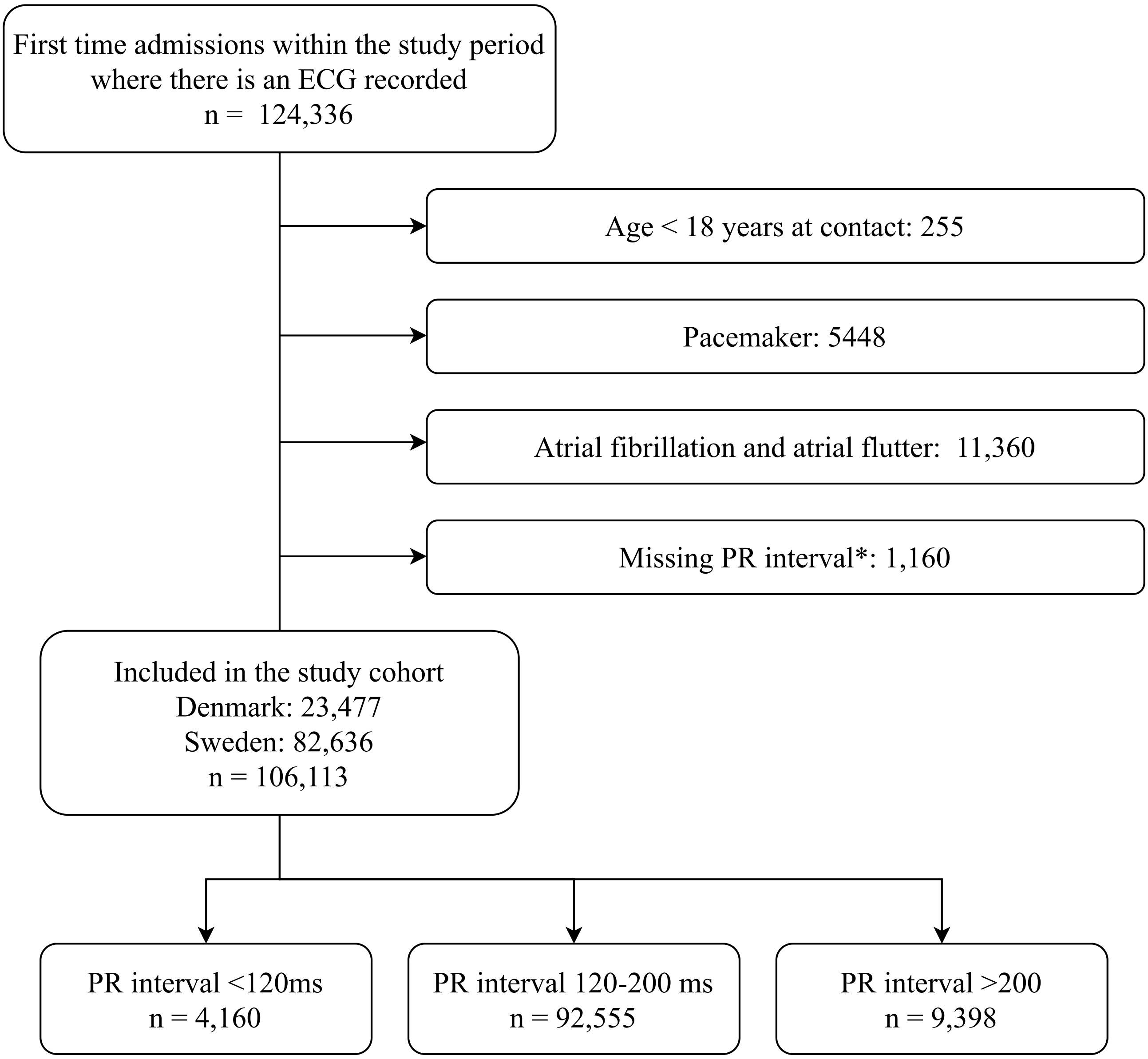

Figure 2: Cubic Spline

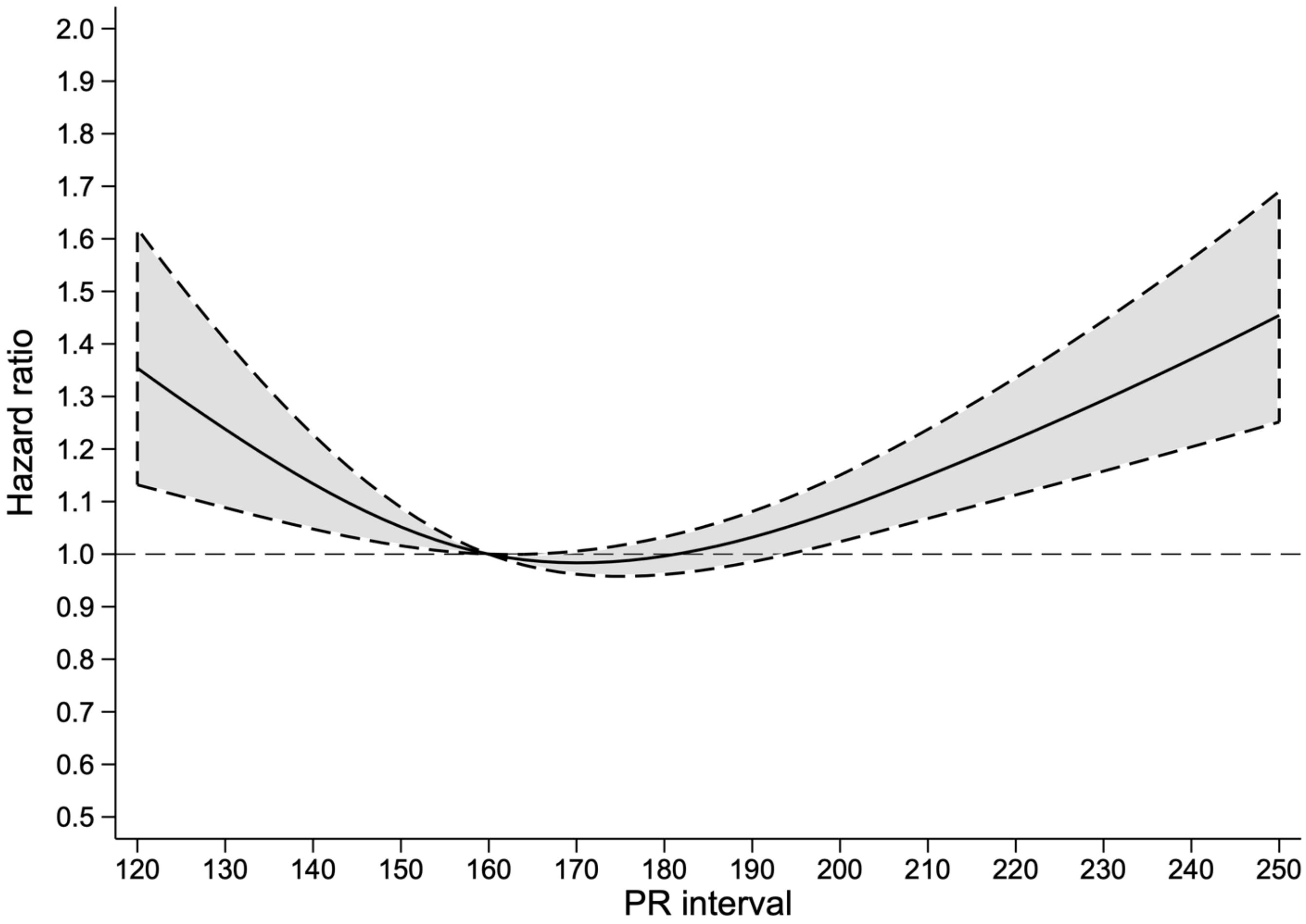

only

Figure 3: Kaplan-Meier

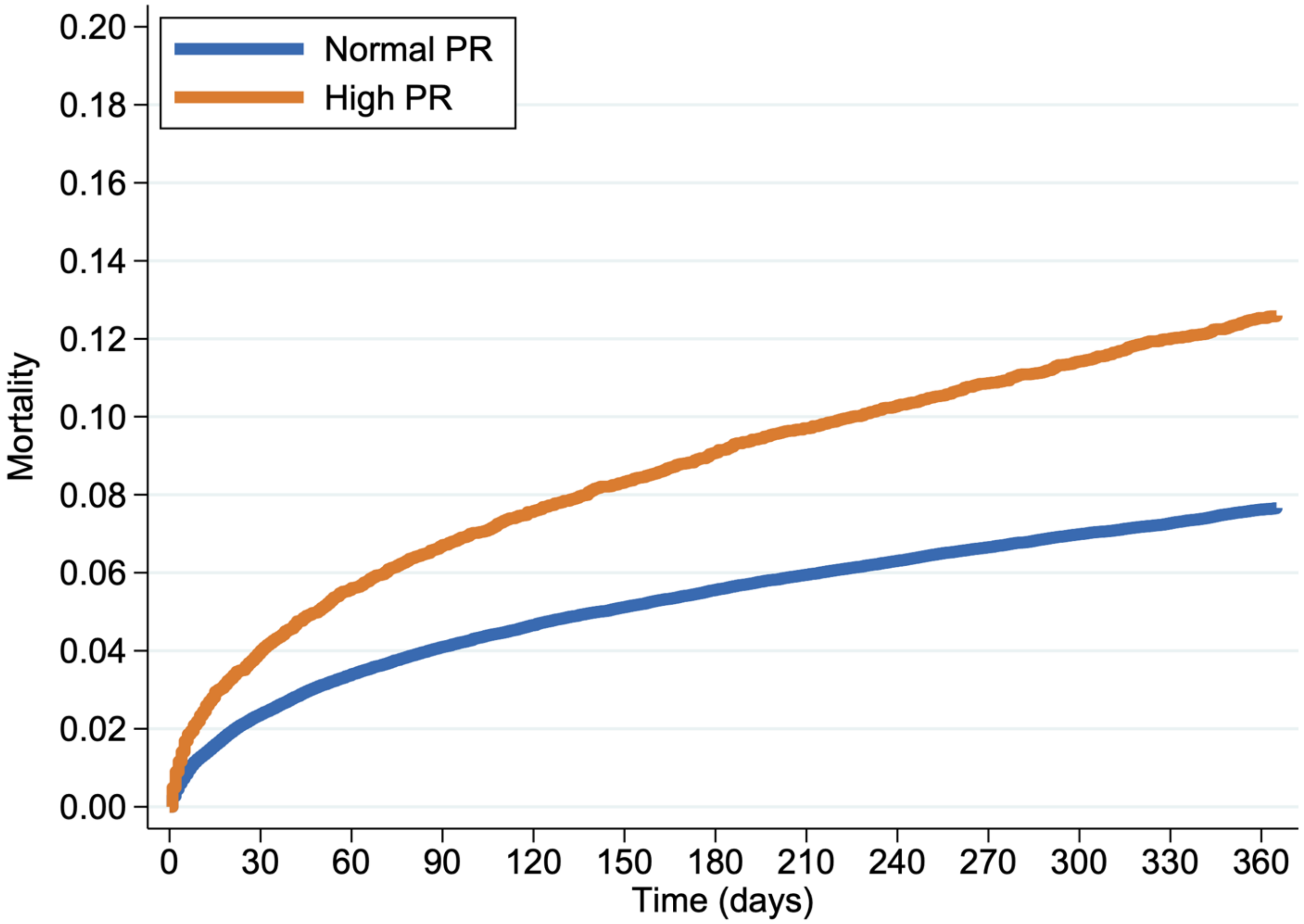

only

Supplementary Appendix

PR interval prolongation and one-year mortality in the emergency department: a transnational multicenter cohort study

Supplementary materials and methods

Appendix A – Data sources

ECGs were extracted from MUSE® Cardiology Information System (GE Healthcare, Wauwatosa, WI, USA) or Megacare Electrocardiographic (ECG) Management System (Simens-Elema, Stockholm, Sweden). Information regarding time of arrival to the ED, triage, course of admission, length of stay, and time of discharge were extracted from the logistic systems in the ED at the Region of Southern Denmark [1] and Region of Skåne.

A unique ten-digit personal civil registration number given in Denmark and Sweden allows individual cross-linkage between databases. Data regarding birth, emigration, and vital status was extracted from the Danish Civil Registration System [2] and the Swedish Population Registry [3].

Data concerning patient comorbidities were retrieved from the Danish National Patient Registry [4] and the Skåne Healthcare Register [5] which contains diagnoses from both primary and secondary healthcare sectors. Data regarding prescriptions came from the Danish National Prescreption Registry [4] and the Swedish National Pharmacy Register [6].

For peer review only

Appendix B – ECG measurements

Danish ECGs were recorded and digitally stored in MUSE® Cardiology Information System (GE Healthcare, Wauwatosa, WI, USA) and processed by the Marquette 12SL algorithm[7]. Swedish ECG's were stored by either Marquette or Philips Diagnostic ECG (Philips) and analysed by the DXL algorithm [8]. ECGs were recorded by pre-hospital medical staff or at either one of the two clinics and all patients who had an ECG recorded within +/- 4 hours of admission to the ED were included. In the case of multiple ECGs recorded on the same individual, the first ECG of acceptable quality was used in the study. Individuals presenting with the following ECGs, obtained by the 12SL algorithm, were excluded from the study: pacemaker or Implantable Cardioverter-Defibrillator (ICD) who had not been excluded by previous criteria (n = 5,448), those with atrial fibrillation and atrial flutter (n = 11,360), and missing PR intervals (n = 1160). Missing PR interval on the ECG was due to bad quality ECG or nodal rhythm on ECG. Pacemaker or ICD were defined as previous history of pacemaker or ICD implantation (Z95.0) and/or pacemaker rhythm recorded on the ECG.

Furthermore, patients presenting with a PR interval <120 ms (n = 643) were excluded from the analysis in order to evaluate PR interval prolongation compared to a normal PR interval of 120-200 ms. The Marquette 12SL algorithm calculates a median PR interval from the 10-second tracing ECG starting at the lead showing the earliest depolarization of the atria to the earliest depolarization of the ventricles in any lead [7]

The ability of the 12SL algorithm to determine PR interval has been evaluated by GE in the Physician's Guide [7] and by Nielsen et al[9], who found a mean difference between manual and 12SL measured PR interval of -0,21 ms (95% CI, -1.17 ms to 0.76 ms) with limits of agreement (±2 standard deviations) ranging from -14.1 ms to 13.7 ms. The ability of the Marquette 12SL algorithm to determine the presence of atrial fibrillation has been validated by Hansen et al [10].

For peer review only

Appendix C – Codes and definitions
C.1: Charlson Comorbidity Index*

Condition	Assigned weight	ICD-10 codes
**Peripheral vascular disease**	1	I70.x, I71x, I73.1, I73.8, I73.9, I77.1, I79.0, I79.2, K55.1, K55.8, K55.9, Z95.8, Z95.9
**Cerebrovascular disease**	1	G45.x, G46.x, H34.0, I60.x-I69.x
**Dementia**	1	F00.x-F03.x, F05.1, G30.x, G31.1
**Chronic pulmonary disease**	1	I27.8, I27.9, J40.x-J47.x, J60.x-J67.x, J68.4, J70.1, J70.3
**Rheumatic disease**	1	M05.x, M06.x, M31.5, M32.x-M34.x, M35.1, M35.3, M36.0
**Peptic ulcer disease**	1	K25.x-K28.x
**Mild liver disease**	1	B18.x, K70.0-K70.3, K70.9, K71.3-K71.5, K71.7, K73.x, K74.x, K76.0, K76.2-K76.4, K76.8, K76.9, Z94.4
**Diabetes without chronic complications**	1	E10.0, E10.1, E10.6, E10.8, E10.9, E11.0, E11.1, E11.6, E11.8, E11.9, E12.0, E12.1, E12.6, E12.8, E12.9, E13.0, E13.1, E13.6, E13.8, E13.9, E14.0, E14.1, E14.6, E14.8, E14.9
**Hemiplegia or paraplegia**	2	G04.1, G11.4, G80.1, G80.2, G81.x, G82.x, G83.0-G83.4, G83.9
**Renal disease**	2	I12.0, I13.1, N03.2-N03.7, N05.2- N05.7, N18.x, N19.x, N25.0, Z49.0-Z49.2, Z94.0, Z99.2
**Diabetes with chronic complications**	2	E10.2-E10.5, E10.7, E11.2-E11.5, E11.7, E12.2-E12.5, E12.7, E13.2- E13.5, E13.7, E14.2-E14.5, E14.7
**Cancer**	2	C00.x-C26.x, C30.x-C34.x, C37.x- C41.x, C43.x, C45.x-C58.x, C60.x-C76.x, C81.x-C85.x, C88.x, C90.x-C97.x
**Metastatic cancer**	3	C77.x-C80.x
**Moderate or severe liver disease**	3	I85.0, I85.9, I86.4, I98.2, K70.4, K71.1, K72.1, K72.9, K76.5, K76.6, K76.7
**AIDS/HIV****	6	B20.x-B22.x, B24.x

**Diagnostic codes for myocardial infarction and heart failure are not included in the index but are included separately in this study as covariates.*
*** AIDS = acquired immunodeficiency syndrome, HIV = human immunodeficiency virus*

C.2: Myocardial infarction and congestive heart failure*

**Myocardial infarction**	I21.x, I22.x, I25.2
**Congestive heart failure**	I09.9, I11.0, I13.0, I13.2, I25.5, I42.0, I42.5-I42.9, I43.x, I50.x, P29.0

** Defined by Charlson Comorbidity Index but included separately in the study as covariates*

C.3: List of drugs associated with PR interval prolongation*

**Beta blocking agents**	C07*
**Calcium channel blockers**	C08*
**Digitalis glycosides**	C01AA*
**Adenosine**	C01EB10

**Usage of drugs are included dichotomously in the analysis defined as a redeemed prescription within 90 days of admission to the emergency department*

For peer review only

Appendix D – Supplementary results

	Study cohort				Matched cohort	
	All	Prolonged PR >200 ms	Normal PR 120-180 ms	LowPR <120 ms	Prolonged PR >200 ms	Normal PR 120-1800 ms
All	(n=92,076)	(n=9,398)	(n=78,518)	(n=4,160)	(n=7,021)	(n=21,063)
Male sex	43,647 (47.4%)	5,554 (59.1%)	36,520 (46.5%)	1,573 (37.8%)	3,631 (51.7%)	10,844 (51.5%)
Age, median (IQR)	60 (42-73)	75 (63-84)	58 (41-71)	52 (34-69)	70 (58-78)	69 (58-78)
18-50	33,707 (36.6%)	1,160 (12.3%)	30,550 (38.9%)	1,997 (48.0%)	1,160 (16.5%)	3,511 (16.7%)
51-69	29,425 (32.0%)	2,372 (25.2%)	25,883 (33.0%)	1,170 (28.1%)	2,342 (33.4%)	7,088 (33.7%)
70+	28,944 (31.4%)	5,866 (62.4%)	22,085 (28.1%)	993 (23.9%)	3,519 (50.1%)	10,464 (49.7%)
Charlson						
0	64,831 (70.4%)	5,787 (61.6%)	56,130 (71.5%)	2,914 (70.0%)	4,554 (64.9%)	13,687 (65.0%)
1	11,999 (13.0%)	1,308 (13.9%)	10,118 (12.9%)	573 (13.8%)	978 (13.9%)	2,961 (14.1%)
2	9,494 (10.3%)	1,496 (15.9%)	7,617 (9.7%)	381 (9.2%)	914 (13.0%)	2,783 (13.2%)
3+	5,752 (6.2%)	807 (8.6%)	4,653 (5.9%)	292 (7.0%)	575 (8.2%)	1,632 (7.7%)
Other diagnoses						
Heart failure	3,650 (4.0%)	949 (10.1%)	2,532 (3.2%)	169 (4.1%)	276 (3.9%)	870 (4.1%)
MI	4,019 (4.4%)	915 (9.7%)	2,965 (3.8%)	139 (3.3%)	359 (5.1%)	1,195 (5.7%)
Use of medication						
QT-prolong drugs	15,277 (16.6%)	2,887 (30.7%)	11,907 (15.2%)	483 (11.6%)	1,601 (22.8%)	4,944 (23.5%)
Center						
Odense	11,850 (12.9%)	1,146 (12.2%)	10,162 (12.9%)	542 (13.0%)	882 (12.6%)	2,646 (12.6%)
South West Jutland	8,559 (9.3%)	932 (9.9%)	7,337 (9.3%)	290 (7.0%)	691 (9.8%)	2,073 (9.8%)
Skåne	37,540 (40.8%)	3,959 (42.1%)	31,686 (40.4%)	1,895 (45.6%)	2,817 (40.1%)	8,451 (40.1%)
Helsingborg	34,127 (37.1%)	3,361 (35.8%)	29,333 (37.4%)	1,433 (34.4%)	2,631 (37.5%)	7,893 (37.5%)
ECG HR, median (IQR)	77 (66-90)	71 (62-83)	77 (67-90)	85 (70-103)	71 (62-83)	78 (67-91)

Supplementary results showing baseline characteristics in patients with normal PR interval (120-199 ms) and severe PR interval prolongation (>200 ms).

References

1. Norgaard B, Mogensen CB, Teglbjaerg LS, et al. Diagnostic packages can be assigned accurately in emergency departments. A multi-centre cohort study. *Dan Med J* 2016;63(6) [published Online First: 2016/06/07]
2. Schmidt M, Pedersen L, Sorensen HT. The Danish Civil Registration System as a tool in epidemiology. *Eur J Epidemiol* 2014;29(8):541-9. doi: 10.1007/s10654-014-9930-3 [published Online First: 2014/06/27]
3. Ludvigsson JF, Otterblad-Olausson P, Pettersson BU, et al. The Swedish personal identity number: possibilities and pitfalls in healthcare and medical research. *Eur J Epidemiol* 2009;24(11):659-67. doi: 10.1007/s10654-009-9350-y [published Online First: 2009/06/09]

For peer review only

4. Schmidt M, Schmidt SA, Sandegaard JL, et al. The Danish National Patient Registry: a review of content, data quality, and research potential. *Clin Epidemiol* 2015;7:449-90. doi: 10.2147/clep.s91125 [published Online First: 2015/11/26]
5. Löfvendahl S, Schelin MEC, Jöud A. The value of the Skåne Health-care Register: Prospectively collected individual-level data for population-based studies. *Scand J Public Health* 2020;48(1):56-63. doi: 10.1177/1403494819868042 [published Online First: 2019/12/10]
6. Astrand B, Hovstadius B, Antonov K, et al. The Swedish National Pharmacy Register. *Stud Health Technol Inform* 2007;129(Pt 1):345-9. [published Online First: 2007/10/04]
7. Marquette 12SL ECG Analysis Program: Physician's Guide. Revision B 2056246-002. https://www.gehealthcare.com: GE Healthcare, 2015:428.
8. Philips DXL ECG Algorithm Physician's Guide. 2 ed. Andover MA: Philips Medical Systems, 2009.
9. Nielsen JB, Pietersen A, Graff C, et al. Risk of atrial fibrillation as a function of the electrocardiographic PR interval: results from the Copenhagen ECG Study. *Heart Rhythm* 2013;10(9):1249-56. doi: 10.1016/j.hrthm.2013.04.012 [published Online First: 2013/04/24]
10. Hansen TG, AP MS, Brandes A, et al. New-onset atrial fibrillation among patients with infection in the emergency department: A multicentre cohort study of one-year stroke risk. *Am J Med* 2019 doi: 10.1016/j.amjmed.2019.06.048 [published Online First: 2019/08/14]

For peer review only

STROBE Statement—Checklist of items that should be included in reports of ***cohort studies***

	Item No	Recommendation	Page No
**Title and abstract**	1	(*a*) Indicate the study's design with a commonly used term in the title or the abstract<br>(*b*) Provide in the abstract an informative and balanced summary of what was done and what was found	1-3
**Introduction**			
Background/rationale	2	Explain the scientific background and rationale for the investigation being reported	4
Objectives	3	State specific objectives, including any prespecified hypotheses	4
**Methods**			
Study design	4	Present key elements of study design early in the paper	5
Setting	5	Describe the setting, locations, and relevant dates, including periods of recruitment, exposure, follow-up, and data collection	5
Participants	6	(*a*) Give the eligibility criteria, and the sources and methods of selection of participants. Describe methods of follow-up<br>(*b*) For matched studies, give matching criteria and number of exposed and unexposed	5
Variables	7	Clearly define all outcomes, exposures, predictors, potential confounders, and effect modifiers. Give diagnostic criteria, if applicable	5-6
Data sources/ measurement	8*	For each variable of interest, give sources of data and details of methods of assessment (measurement). Describe comparability of assessment methods if there is more than one group	5-6
Bias	9	Describe any efforts to address potential sources of bias	6
Study size	10	Explain how the study size was arrived at	5-6
Quantitative variables	11	Explain how quantitative variables were handled in the analyses. If applicable, describe which groupings were chosen and why	6
Statistical methods	12	(*a*) Describe all statistical methods, including those used to control for confounding<br>(*b*) Describe any methods used to examine subgroups and interactions<br>(*c*) Explain how missing data were addressed<br>(*d*) If applicable, explain how loss to follow-up was addressed<br>(*e*) Describe any sensitivity analyses	6-7
**Results**			
Participants	13*	(a) Report numbers of individuals at each stage of study—eg numbers potentially eligible, examined for eligibility, confirmed eligible, included in the study, completing follow-up, and analysed<br>(b) Give reasons for non-participation at each stage<br>(c) Consider use of a flow diagram	8
Descriptive data	14*	(a) Give characteristics of study participants (eg demographic, clinical, social) and information on exposures and potential confounders<br>(b) Indicate number of participants with missing data for each variable of interest<br>(c) Summarise follow-up time (eg, average and total amount)	8
Outcome data	15*	Report numbers of outcome events or summary measures over time	8

For peer review only - http://bmjopen.bmj.com/site/about/guidelines.xhtml

For peer review only

Main results	16	(*a*) Give unadjusted estimates and, if applicable, confounder-adjusted estimates and their precision (eg, 95% confidence interval). Make clear which confounders were adjusted for and why they were included<br>(*b*) Report category boundaries when continuous variables were categorized<br>(*c*) If relevant, consider translating estimates of relative risk into absolute risk for a meaningful time period	8
Other analyses	17	Report other analyses done—eg analyses of subgroups and interactions, and sensitivity analyses	8
**Discussion**			
Key results	18	Summarise key results with reference to study objectives	8-9
Limitations	19	Discuss limitations of the study, taking into account sources of potential bias or imprecision. Discuss both direction and magnitude of any potential bias	9-10
Interpretation	20	Give a cautious overall interpretation of results considering objectives, limitations, multiplicity of analyses, results from similar studies, and other relevant evidence	9-10
Generalisability	21	Discuss the generalisability (external validity) of the study results	9-10
**Other information**			
Funding	22	Give the source of funding and the role of the funders for the present study and, if applicable, for the original study on which the present article is based	1

*Give information separately for exposed and unexposed groups.

Note: An Explanation and Elaboration article discusses each checklist item and gives methodological background and published examples of transparent reporting. The STROBE checklist is best used in conjunction with this article (freely available on the Web sites of PLoS Medicine at http://www.plosmedicine.org/, Annals of Internal Medicine at http://www.annals.org/, and Epidemiology at http://www.epidem.com/). Information on the STROBE Initiative is available at http://www.strobe-statement.org.

For peer review only

For peer review only - http://bmjopen.bmj.com/site/about/guidelines.xhtml

PR interval prolongation and one-year mortality among emergency department patients: a multicenter transnational cohort study

Journal:	*BMJ Open*
Manuscript ID	bmjopen-2021-054238.R1
Article Type:	Original research
Date Submitted by the Author:	25-Oct-2021

Complete List of Authors:	Pedersen, Rune; Odense University Hospital, 1. Department of Emergency Medicine<br>Larsen, Tobias; Odense University Hospital, Department of Emergency Medicine<br>Jensen, Helene; University of Southern Denmark, Clinical Pharmacology, Pharmacy, and Environmental Medicine, Department of Public Health,<br>Brabrand, Mikkel; Southwest Jutland Hospital Esbjerg, Department of Emergency Medicine<br>Lundager Forberg, Jakob; Helsingborgs lasarett, Department of Emergency Medicine and Prehospital Care<br>Ekelund, U; Lunds Universitet, Department of Emergency Medicine<br>Pottegard, Anton; University of Southern Denmark, Clinical Pharmacology, Pharmacy, and Environmental Medicine, Department of Public Health<br>Lassen, Annmarie; Odense University Hospital, Department of Emergency Medicine

**Primary Subject Heading**:	Emergency medicine
Secondary Subject Heading:	Epidemiology, Cardiovascular medicine
Keywords:	ACCIDENT & EMERGENCY MEDICINE, Cardiac Epidemiology < CARDIOLOGY, EPIDEMIOLOGY

SCHOLARONE™
Manuscripts

I, the Submitting Author has the right to grant and does grant on behalf of all authors of the Work (as defined in the below author licence), an exclusive licence and/or a non-exclusive licence for contributions from authors who are: i) UK Crown employees; ii) where BMJ has agreed a CC-BY licence shall apply, and/or iii) in accordance with the terms applicable for US Federal Government officers or employees acting as part of their official duties; on a worldwide, perpetual, irrevocable, royalty-free basis to BMJ Publishing Group Ltd ("BMJ") its licensees and where the relevant Journal is co-owned by BMJ to the co-owners of the Journal, to publish the Work in this journal and any other BMJ products and to exploit all rights, as set out in our licence.

The Submitting Author accepts and understands that any supply made under these terms is made by BMJ to the Submitting Author unless you are acting as an employee on behalf of your employer or a postgraduate student of an affiliated institution which is paying any applicable article publishing charge ("APC") for Open Access articles. Where the Submitting Author wishes to make the Work available on an Open Access basis (and intends to pay the relevant APC), the terms of reuse of such Open Access shall be governed by a Creative Commons licence – details of these licences and which Creative Commons licence will apply to this Work are set out in our licence referred to above.

Other than as permitted in any relevant BMJ Author's Self Archiving Policies, I confirm this Work has not been accepted for publication elsewhere, is not being considered for publication elsewhere and does not duplicate material already published. I confirm all authors consent to publication of this Work and authorise the granting of this licence.

For peer review only

PR interval prolongation and one-year mortality among emergency department patients: a multicenter transnational cohort study

Rune Vad [CO, 1]
Tobias Malte Larsen [CO, 1]
Helene Kildegaard[2], Mikkel Brabrand[1,3], Jakob Lundager Forberg[4], Ulf Ekelund[5], Anton Pottegård[2], Annmarie Touborg Lassen[1]

Author affiliations:

1. Department of Emergency Medicine, Odense University Hospital, Odense, Denmark
2. Clinical Pharmacology, Pharmacy, and Environmental Medicine, Department of Public Health, University of Southern Denmark, Odense, Denmark
3. Department of Emergency Medicine, Hospital of South West Jutland, Esbjerg, Denmark
4. Department of Emergency Medicine, Helsingborg Hospital, Helsingborg, Sweden
5. Department of Emergency Medicine, Skåne University Hospital, Lund, Sweden

Corresponding author

Rune Vad Pedersen

Hjallesevej 54 2.sal, 5230 Odense M

rune@vadpedersen.dk

Tel. 0045 30296343

Word count: 2317

Abstract word count: 229

For peer review only

Abstract

Objectives

Emerging evidence supports that PR interval prolongation is associated with increased mortality. However, most previous studies have limited confounder control, and clinical impact in a population of acute ill patients is unknown. The aim of this study was to investigate whether one-year all-cause mortality was increased in patients presenting with PR interval prolongation in the emergency department (ED).

Design and setting

We conducted a register-based cohort study in two Swedish and two Danish EDs. We included all adult patients with an ECG performed at arrival to the Danish EDs during March 2013 to May 2014 and Swedish EDs during January 2010 to January 2011. Using propensity score matching, we analyzed hazard ratio (HR) for one-year all-cause mortality comparing patients with PR interval prolongation (>200 ms) and normal PR interval (120-200 ms).

Participants and results

We included 106,124 patients. PR interval prolongation occurred in 8.9% (95% CI, 8.7-9.0); these patients were older and had more co-morbidity than those with a normal PR interval. The absolute one-year risk of death was 13% (95% CI, 12.3-13.7) for patients with PR interval prolongation and 7.9% (95% CI 7.7-8.0) for those without. After confounder adjustments by propensity score matching, PR interval prolongation showed no association with one-year mortality with a HR of 1.00 (95% CI, 0.93-1.08).

Conclusion

PR interval prolongation does not constitute an independent risk factor for one-year mortality in ED patients.

For peer review only

Strengths and limitations

- Large multicenter transnational study population
- Cross-linkage of several databases
- Comprehensive statistical analysis including a propensity score matched cohort
- Due to the nature of the study design causality cannot be assessed
- No stratification by cause of admission to the ED

Introduction

As a measure of conduction time from the onset of atrial depolarization to the beginning of ventricular depolarization, the PR interval reflects the propagation of electrical impulses from the sinus node to the ventricles. A delay in the propagation of electrical impulses resulting in PR interval prolongation (>200 ms) is clinically known as first-degree atrioventricular block or delay.[1] PR interval prolongation has clinically been considered a benign condition, but recent studies provide increasing evidence that PR interval prolongation in different populations is a predictor of future atrial fibrillation, implantation of a pacemaker or an implantable cardioverter-defibrillator, major cardiac events, and all-cause mortality.[2] The most broadly accepted pathogenetic explanation of a possible association between PR interval prolongation and mortality is that age-related myocardial fibrosis plays a role in delayed electric conduction and increased vulnerability to arrhythmia.[3]

The subject is still debated, and the clinical consequence of a possible increased risk is unclear. Most prior studies have been performed in healthy populations with 5-35 years of follow-up.[4-6] No previous studies have evaluated the prognostic significance of PR interval prolongation among acutely ill patients.

In this study, we hypothesized that PR interval prolongation is associated with an increased risk of one-year all-cause mortality in patients presenting to the emergency department (ED).

For peer review only - http://bmjopen.bmj.com/site/about/guidelines.xhtml

Materials and methods

Study design and setting

This was a register-based multi-centre cohort study based on adult ED patients in the two Danish hospitals Odense University Hospital (OUH) and Hospital of South West Jutland (SVS) and the two Swedish hospitals Skåne University Hospital (SUS) at Lund and Helsingborg Hospital (HH). The study was based on Danish data from 1st of March 2013 to 1st of May 2014 and Swedish data from 1st of January 2010 to 1st of January 2011. OUH covers a population of 270,000 people while SVS covers a population of 220,000 people. SUS Lund covers 270,000 people while the regional hospital HH covers 250,000.

In Denmark and Sweden, the health care system is tax-funded; hence, all residents have free access to healthcare, though with a small co-payment in Sweden.

Selection of patients

All adult patients (≥ 18 years), who had a 12-lead ECG recorded within 4 hours of arrival to the ED, were eligible for the study. In case of multiple recordings, the first ECG taken within this period was used. Patients with multiple contacts within the study period were included only at their first contact. Contacts with invalid Civil Personal Registration (CPR) number and patients aged <18 years were excluded. Patients with loss to follow-up due to emigration were censored. Furthermore, patients with a pacemaker, atrial fibrillation or flutter, and missing PR intervals were excluded (Figure 1).

Data sources

A unique CPR number is given to every resident in Denmark and Sweden and used in all hospital contacts, which allows individual cross-linkage between databases. ECGs were extracted from electronic central databases at the Region of Southern Denmark and Region of Skåne. Logistic information was extracted from the logistic systems at the EDs of the Region of Skåne and Region of Southern Denmark.[7] Patient comorbidities were retrieved from the Danish National Patient Registry[8] and the Skåne Healthcare Databases, data regarding prescriptions from the Danish National Prescription Registry[9] and the Swedish Pharmacy Registry,[10] and data regarding birth,

For peer review only - http://bmjopen.bmj.com/site/about/guidelines.xhtml

emigration, and vital status were obtained from the Danish Civil Registration System[11] and the Swedish Population Register.[12] Appendix A provides further details regarding data sources.

Definitions and ECG measurements

A normal PR interval was defined as 120-200 ms.[1] PR interval prolongation was defined as PR interval > 200 ms.[1] The PR interval was automatically calculated as a median value by the GE Marquette 12SL ECG Analysis Program or Philips DXL and stored in the MUSE Cardiology Information System or the Megacare Electrocardiographic (ECG) Management System (Simens-Elema, Stockholm, Sweden), respectively. The ability of the Marquette 12SL algorithm to determine PR interval has been evaluated by GE[13] and by Nielsen et al.[14] who found a mean difference of -0.21 ms (95% CI: -1.17 - 0.76 ms) compared to manual measurement. Appendix B provides further information on ECG measurements and validation.

Comorbidity status was defined by the Charlson Comorbidity Index (CCI,[15 16]) with exclusion of myocardial infarction (MI) and congestive heart failure (CHF) which were used as separate covariates in the propensity score matching. Appendix C.1 provides further information on CCI and appendix C.2 on MI and CHF. Usage of PR interval prolonging drugs was defined as redeemed prescriptions of these drugs within the last 90 days (appendix C.3).

Statistics and primary analysis

Continuous data are presented as medians with interquartile ranges (IQR) and categorical data as proportions with 95% CIs based on a binomial distribution. The mortality of patients with PR interval prolongation was compared with patients having a normal PR interval.

Patients were followed from ED visit until death or 365 days, whichever came first. Survival was represented by a Kaplan-Meier survival curve. Furthermore, the crude relationship between PR interval and one-year all-cause mortality was illustrated using restricted cubic splines.[17] Unadjusted one-year all-cause mortality was presented as absolute risk and assessed using univariate Cox regression comparing patients with PR interval prolongation (>200 ms) and normal PR interval (120-200 ms). Propensity scores were estimated for each individual by use of logistic regression with a PR interval of > 200 ms as a binary outcome. The following covariates were included in the logistic regression model: sex, age, CCI, myocardial infarction, congestive heart failure, study centre, and PR interval prolonging drugs. We also present a model including only age and sex as covariates. We generated a propensity matched cohort by a 1:3 parallel, balanced, nearest neighbor

For peer review only - http://bmjopen.bmj.com/site/about/guidelines.xhtml

matching with a caliper of 0.02, and estimated hazards ratios for one-year mortality using Cox regression. Heart rate was adjusted for after propensity score matching by multivariate regression.

All statistical tests were 2-sided with p-values < 0.05 considered statistically significant. Stata version 16 (StataCorp, College Station, TX, USA) was used for analyses.

Sensitivity analyses

30 days mortality was calculated based on the propensity score matched cohort. As a sensitivity analysis, a second propensity score matched cohort was created after exclusion of patients with PR intervals between 180 and 199.

Ethics

The study was approved by Region of Southern Denmark (Journal nr. 19/16024) and the Danish Health Authority (No. 3-3013-1031). In accordance with Swedish law, the study was approved by the regional ethics review board at Lund and by Region Skåne (Dnr 2015/129). The data were anonymized before being accessed by the authors.

Patient and public involvement

This was a study without direct patient contact.

For peer review only

Results

We identified 124,336 ED contacts within the study period. A total of 106,124 patients were included in the study. Reasons for exclusion are presented in Figure 1. Among patients included in the study, PR interval prolongation (>200 ms) occurred in 9,397 (9%) patients. Among those with PR interval prolongation, the average age was 75 years and 59% were male. Patients with PR interval prolongation were generally older, more likely to be male, had more comorbidities, and took more medications than those with a normal PR interval (Table 1).

In the propensity score matched cohort, 32,952 patients were included. A total of 8,238 patients with PR interval prolongation were matched with 24,714 patients with a normal PR interval (Table 1).

Prognosis

With 8,830 deaths in the cohort, one-year all-cause mortality was 8.3% (95% CI: 8.2 - 8.5). The cubic spline showed a U-shaped association between PR interval and mortality (Figure 2).

Among 9,397 patients with PR interval prolongation, 1,219 died, representing a 13% (95% CI,12.3 - 13.7) absolute one-year risk of death. Among 96,727 patients with normal PR interval, 7,611 died representing an 7.9 % (95% CI: 7.7 - 8.0) absolute risk of death. The unadjusted relative risk of death given PR interval prolongation was 1.65 (95% CI: 1.56 - 1.75; Figure 3).

In the heart rate adjusted propensity score matched cohort, PR interval prolongation had a HR of 1.00 (95% CI 0.93 - 1.08**)** for one-year mortality (Table 2) and a HR of 1.11 (95% CI 0.97 - 1.28) for 30-day all-cause mortality. When adjusting only for sex and age PR interval prolongation had a HR of 0.96 (95% CI 0.89 – 1.03)(Appendix D.1).

Sensitivity analyses

After exclusion of PR intervals ranging from 180-199 ms, patients with PR interval prolongation had a HR of 0.90 (95% CI: 0.82 - 0.99) for one-year mortality (Table 2). Appendix D.2 provides supplementary baseline characteristics.

Post hoc analysis

To investigate the association between PR interval prolongation and adverse cardiovascular events we have conducted the same analysis with myocardial infarction (MI) and congestive heart failure (CHF) as outcomes. This showed that PR interval prolongation is associated with an increased risk

For peer review only - http://bmjopen.bmj.com/site/about/guidelines.xhtml

of MI and CHF with a hazard ratio of 1.26 (95% CI 1.07 - 1.49 and 1.16 (95% CO 1.01 - 1.34), respectivly. Apendix E provides further information.

Discussion

With a focus on one-year all-cause mortality, the present study was designed to investigate the transferability of results from prior studies onto acutely ill patients. We found that PR interval prolongation did not constitute an independent risk factor for one-year all-cause mortality among unselected ED patients. This result corresponds to the clinical practice where no interventions are needed with regards to asymptomatic patients with first-degree atrioventricular block.[1]

Our result differs from previous studies in healthy populations which suggest a possible association between prolonged PR interval and significant increases in atrial fibrillation and mortality.[2] The diverging results may be due to important differences in study design and population. Most previous studies of prolonged PR interval provide information of 5-35 years mortality,[2 4 18] whereas this is the first study of PR interval prolongation and prognosis in an ED setting. The unique civil registration number allowed cross-linkage between different databases which resulted in very little loss to follow-up. Hazard ratios were calculated on the heart rate adjusted propensity score matched cohort, and this represents a thorough confounder control which contributes to the robustness of the statistical analysis. Few prior studies[18 19] have included this level of confounder control. One could hypothesize that underlying cardiovascular diseases is linked with PR interval prolongation and matching our cohort for underlying cardiovascular diseases would contribute to a lack of association. Supplementary appendix D.1 provides an analysis only adjusted for age and sex and shows no association between PR interval prolongation and death. This proves that the increased unadjusted absolute risk of death is driven by sex and age and not by underlying cardiovascular diseases.

We also conducted a sensitivity analysis using a "twilight" which excluded individuals with a PR interval of 180-199 ms and supported our main analysis. Our results thus indicate that the increased unadjusted relative risk is due to confounders and not PR interval prolongation. Inclusion of patients from four centers, two tertiary referral centers (OUH and SUS) and two regional hospitals (SVS and HH), strengthen the external validity. Further, a relatively large study population compared to prior studies[4 6 18 20] improves the precision of the results.

For peer review only - http://bmjopen.bmj.com/site/about/guidelines.xhtml

In our study we found an increased absolute risk of one-year mortality in patients with very short as well as prolonged PR interval (Figure 2). On an individual patient level, the unadjusted absolute risk of one-year death was increased by 65% given a PR interval prolongation. The correlation between a higher heart rate and shorter PR interval may explain the increased probability of death among patients with short PR interval. Furthermore, diseases with ventricular pre-excitation cause shortening of the PR interval and might also contribute, but pre-excitatory syndromes like Wolff-Parkinson-White are very rare, and are unlikely to contribute to the present observation.[21] In the main analysis heart rate was adjusted for after propensity score matching, and thereby did not influence the results.

The present study has some limitations. An observational study cannot investigate causality but only test and generate hypotheses. Though propensity score matching has some strengths, it does not balance unmeasured confounders. Of note, BMI, smoking status, blood pressure, ethnicity, and socioeconomic status were not possible to extract from the database.

Further, an ECG performed in the ED is just a snapshot, as the PR interval could have been different just hours earlier and it is therefore unknown whether PR interval prolongation regress, persists, or develops into more severe cardiac disease. Also, the PR interval varies with circadian rhythm.[22] Although this variation could result in misclassification, it is expected to distribute evenly between patients with prolonged and normal PR interval.

We did not analyze the hazards in different patient subgroups, e.g. based on chief complaint at the ED or comorbidities. In this context, it is reasonable to believe that PR interval prolongation may be a better predictor for adverse events in individuals presenting with syncope, presyncope, palpitations etc.[23] It is unclear whether these patients would benefit from closer monitoring, e.g. with an ECG every 2-5 years, as previously suggested.[2]

However, in a post hoc analysis we investigated the association between prolonged PR interval and the one-year risk of MI and CHF. This showed a small but significant increased risk of these cardiovascular events in patients presenting with PR interval prolongation (Appendix E). This result strengthens the theory that first degree heart block is linked with adverse cardiovascular events. Though it is a major limitation to the post hoc analysis that the cohort does not include important risk factors such as smoking status, familiar dispositions and cholesterol-levels.

For peer review only

The population in Denmark and Sweden have a universal access to the healthcare system. Generalization of our results outside of Western Europe is therefore uncertain. Another limitation is that most of the patients in the present study are Caucasians. PR interval has been shown to be a more sensitive predictor of atrial fibrillation among African Americans.[24]

In conclusion, PR interval prolongation did not constitute an independent risk factor for one-year all-cause mortality among unselected ED patients.

For peer review only

For peer review only - http://bmjopen.bmj.com/site/about/guidelines.xhtml

Contributors

Co-First/Equal authorship: TML and RVP contributed equally to this work. TML, RVP, AP and AL designed the study, interpreted the results and TML and RVP drafted the paper. TML, RVP, AP and HJ analyzed the data. HJ, UE, ATL, MB, and JLF created the EXPECT database. All authors critically reviewed the paper, assisted with interpretation of the results and approved the final edition.

Conflicts of interest

The authors declare no conflicts of interest.

Data sharing

Due to the danish law on personal data, we are not allowed to share data in a public dataset. All data relevant to the study are included in the article or uploaded as supplementary information.

Funding

The work was supported by Odense University Hospital and the Swedish heart-Lung Foundation. This study was also part of the AIR Lund (Artificially Intelligent use of Resisters at Lund University) research environment and received funding from the Swedish Research Council (VR; grant no. 2019-00198).

For peer review only

References

1. Kusumoto FM, Schoenfeld MH, Barrett C, et al. 2018 ACC/AHA/HRS Guideline on the Evaluation and Management of Patients With Bradycardia and Cardiac Conduction Delay. *Circulation* 2018:Cir0000000000000628. doi: 10.1161/cir.0000000000000628 [published Online First: 2018/12/28]
2. Kwok CS, Rashid M, Beynon R, et al. Prolonged PR interval, first-degree heart block and adverse cardiovascular outcomes: a systematic review and meta-analysis. *Heart (British Cardiac Society)* 2016;102(9):672-80. doi: 10.1136/heartjnl-2015-308956 [published Online First: 2016/02/18]
3. de Jong S, van Veen TA, van Rijen HV, et al. Fibrosis and cardiac arrhythmias. *J Cardiovasc Pharmacol* 2011;57(6):630-8. doi: 10.1097/FJC.0b013e318207a35f [published Online First: 2010/12/15]
4. Aro AL, Anttonen O, Kerola T, et al. Prognostic significance of prolonged PR interval in the general population. *European heart journal* 2014;35(2):123-9. doi: 10.1093/eurheartj/eht176 [published Online First: 2013/05/17]
5. Chan YH, Hai JJ, Lau KK, et al. PR interval prolongation in coronary patients or risk equivalent: excess risk of ischemic stroke and vascular pathophysiological insights. *BMC Cardiovasc Disord* 2017;17(1):233. doi: 10.1186/s12872-017-0667-2 [published Online First: 2017/08/25]
6. Magnani JW, Wang N, Nelson KP, et al. Electrocardiographic PR interval and adverse outcomes in older adults: the Health, Aging, and Body Composition study. *Circ Arrhythm Electrophysiol* 2013;6(1):84-90. doi: 10.1161/circep.112.975342 [published Online First: 2012/12/18]
7. Norgaard B, Mogensen CB, Teglbjaerg LS, et al. Diagnostic packages can be assigned accurately in emergency departments. A multi-centre cohort study. *Dan Med J* 2016;63(6) [published Online First: 2016/06/07]
8. Schmidt M, Schmidt SA, Sandegaard JL, et al. The Danish National Patient Registry: a review of content, data quality, and research potential. *Clin Epidemiol* 2015;7:449-90. doi: 10.2147/clep.s91125 [published Online First: 2015/11/26]
9. Pottegård A, Hallas J, Sørensen HT, et al. Data Resource Profile: The Danish National Prescription Registry. *International Journal of Epidemiology* 2016;46(3):798-98f. doi: 10.1093/ije/dyw213
10. Astrand B, Hovstadius B, Antonov K, et al. The Swedish National Pharmacy Register. *Stud Health Technol Inform* 2007;129(Pt 1):345-9. [published Online First: 2007/10/04]
11. Schmidt M, Pedersen L, Sorensen HT. The Danish Civil Registration System as a tool in epidemiology. *Eur J Epidemiol* 2014;29(8):541-9. doi: 10.1007/s10654-014-9930-3 [published Online First: 2014/06/27]
12. Ludvigsson JF, Otterblad-Olausson P, Pettersson BU, et al. The Swedish personal identity number: possibilities and pitfalls in healthcare and medical research. *Eur J Epidemiol* 2009;24(11):659-67. doi: 10.1007/s10654-009-9350-y [published Online First: 2009/06/09]
13. Marquette 12SL ECG Analysis Program: Physician's Guide. Revision B 2056246-002. https://www.gehealthcare.com: GE Healthcare, 2015:428.

For peer review only

14. Nielsen JB, Pietersen A, Graff C, et al. Risk of atrial fibrillation as a function of the electrocardiographic PR interval: results from the Copenhagen ECG Study. *Heart Rhythm* 2013;10(9):1249-56. doi: 10.1016/j.hrthm.2013.04.012 [published Online First: 2013/04/24]
15. Quan H, Sundararajan V, Halfon P, et al. Coding algorithms for defining comorbidities in ICD-9-CM and ICD-10 administrative data. *Med Care* 2005;43(11):1130-9. doi: 10.1097/01.mlr.0000182534.19832.83 [published Online First: 2005/10/15]
16. Charlson ME, Pompei P, Ales KL, et al. A new method of classifying prognostic comorbidity in longitudinal studies: development and validation. *J Chronic Dis* 1987;40(5):373-83. doi: 10.1016/0021-9681(87)90171-8 [published Online First: 1987/01/01]
17. Durrleman S, Simon R. Flexible regression models with cubic splines. *Stat Med* 1989;8(5):551-61. doi: 10.1002/sim.4780080504 [published Online First: 1989/05/01]
18. Cheng S, Keyes MJ, Larson MG, et al. Long-term outcomes in individuals with prolonged PR interval or first-degree atrioventricular block. *Jama* 2009;301(24):2571-7. doi: 10.1001/jama.2009.888 [published Online First: 2009/06/25]
19. Aro AL, Anttonen O, Kerola T, et al. Prognostic significance of prolonged PR interval in the general population. *European heart journal* 2013;35(2):123-29. doi: 10.1093/eurheartj/eht176
20. Hisamatsu T, Miura K, Fujiyoshi A, et al. Long-term outcomes associated with prolonged PR interval in the general Japanese population. *Int J Cardiol* 2015;184:291-3. doi: 10.1016/j.ijcard.2015.02.028 [published Online First: 2015/03/04]
21. Mark DG, Brady WJ, Pines JM. Preexcitation syndromes: diagnostic consideration in the ED. *Am J Emerg Med* 2009;27(7):878-88. doi: 10.1016/j.ajem.2008.06.013 [published Online First: 2009/08/18]
22. Dilaveris PE, Farbom P, Batchvarov V, et al. Circadian behavior of P-wave duration, P-wave area, and PR interval in healthy subjects. *Ann Noninvasive Electrocardiol* 2001;6(2):92-7. [published Online First: 2001/05/03]
23. Lewalter T, Purerfellner H, Ungar A, et al. "First-degree AV block-a benign entity?" Insertable cardiac monitor in patients with 1st-degree AV block reveals presence or progression to higher grade block or bradycardia requiring pacemaker implant. *J Interv Card Electrophysiol* 2018;52(3):303-06. doi: 10.1007/s10840-018-0439-7 [published Online First: 2018/08/15]
24. Shulman E, Aagaard P, Kargoli F, et al. Validation of PR interval length as a criterion for development of atrial fibrillation in non-Hispanic whites, African Americans and Hispanics. *J Electrocardiol* 2015;48(4):703-9. doi: 10.1016/j.jelectrocard.2015.04.015 [published Online First: 2015/05/31]

For peer review only - http://bmjopen.bmj.com/site/about/guidelines.xhtml

Tables and figures:

Table 1: Baseline characteristics

	Study cohort				Matched cohort	
	All	Prolonged PR >200 ms	Normal PR 120-200 ms	Short PR <120 ms	Prolonged PR >200 ms	Normal PR 120-200 ms
All	(n=106,124)	(n=9,397)	(n=92,565)	(n=4,162)	(n=8,238)	(n=24,714)
Male sex	51,156 (48.2%)	5,558 (59.1%)	44,029 (47.6%)	1,569 (37.7%)	4,569 (55.5%)	13,710 (55.5%)
Age, median (IQR)	61 (43-74)	75 (63-84)	59 (43-73)	52 (34-69)	72 (61-81)	72 (60-81)
18-50	36,748 (34.6%)	1,157 (12.3%)	33,592 (36.3%)	1,999 (48.0%)	1,157 (14.0%)	3,494 (14.1%)
51-69	34,165 (32.2%)	2,373 (25.3%)	30,621 (33.1%)	1,171 (28.1%)	2,371 (28.8%)	7,250 (29.3%)
70+	35,211 (33.2%)	5,867 (62.4%)	28,352 (30.6%)	992 (23.8%)	4,710 (57.2%)	13,970 (56.5%)
Charlson						
0	74,396 (70.1%)	5,785 (61.6%)	65,693 (71.0%)	2,918 (70.1%)	5,186 (63.0%)	15,667 (63.4%)
1	13,750 (13.0%)	1,309 (13.9%)	11,866 (12.8%)	575 (13.8%)	1,154 (14.0%)	3,415 (13.8%)
2	11,239 (10.6%)	1,494 (15.9%)	9,366 (10.1%)	379 (9.1%)	1,196 (14.5%)	3,591 (14.5%)
3+	6,739 (6.4%)	809 (8.6%)	5,640 (6.1%)	290 (7.0%)	702 (8.5%)	2,041 (8.3%)
Other diagnoses						
Heart failure	4,473 (4.2%)	950 (10.1%)	3,356 (3.6%)	167 (4.0%)	509 (6.2%)	1,640 (6.6%)
MI	4,933 (4.6%)	913 (9.7%)	3,881 (4.2%)	139 (3.3%)	590 (7.2%)	1,870 (7.6%)
Use of medication						
QT-prolong drugs	18,560 (17.5%)	2,886 (30.7%)	15,191 (16.4%)	483 (11.6%)	2,195 (26.6%)	6,750 (27.3%)
Center						
Odense	13,572 (12.8%)	1,146 (12.2%)	11,884 (12.8%)	542 (13.0%)	980 (11.9%)	2,940 (11.9%)
South West Jutland	9,905 (9.3%)	932 (9.9%)	8,683 (9.4%)	290 (7.0%)	815 (9.9%)	2,445 (9.9%)
Skåne	43,616 (41.1%)	3,960 (42.1%)	37,757 (40.8%)	1,899 (45.6%)	3,376 (41.0%)	10,128 (41.0%)
Helsingborg	39,031 (36.8%)	3,359 (35.7%)	34,241 (37.0%)	1,431 (34.4%)	3,067 (37.2%)	9,201 (37.2%)
ECG HR, median (IQR)	76 (66-89)	71 (62-83)	76 (66-90)	85 (70-103)	71 (62-83)	77 (66-90)

*Table 1 Baseline characteristics of the study cohort and the propensity score matched cohort. *PR = PR interval*

For peer review only

For peer review only - http://bmjopen.bmj.com/site/about/guidelines.xhtml

Table 2: Risk assessment in the study population

	n	Events	HR (CI 95%)
Propensity score matched cohort*			
One-year all-cause mortality			
Normal PR 120-200 ms	24,753	3,129	1.0 (ref)
Prolonged PR >200 ms	8,251	895	1.00 (0.93-1.08)
30 days all-cause mortality			
Normal PR 120-200 ms	24,753	1,042	1.0 (ref)
Prolonged PR >200 ms	8,251	308	1.11 (0.97-1.28)
Sensitivity analysis**			
One-year all-cause mortality			
Normal PR 120-180 ms	21,063	2,367	1.0 (ref)
Prolonged PR >200 ms	7,021	606	0.90 (0.82-0.99)

*Table 2 Risk assessment in the study population. *Heart rate adjusted propensity score matched cohort. **Sensitivity analysis on heart rate adjusted propensity score matched cohort excluding PR intervals between 180-199 ms (supplementary D)*

Figure 1: Flow chart of the study population. ECG = electrocardiogram; * arrhythmias not compatible with PR interval measurement

Figure 2: Restricted cubic spline analysis. Estimated probability of one-year all-cause mortality presented as a function of PR interval duration ranging from 120 to 250. 95% CI limits illustrated by the shaded area.

Figure 3: Kaplan-Meier survival curve displaying the survival of patients with a normal PR interval of 120-200ms (blue) and patients with a prolonged PR interval of > 200ms (orange)

For peer review only

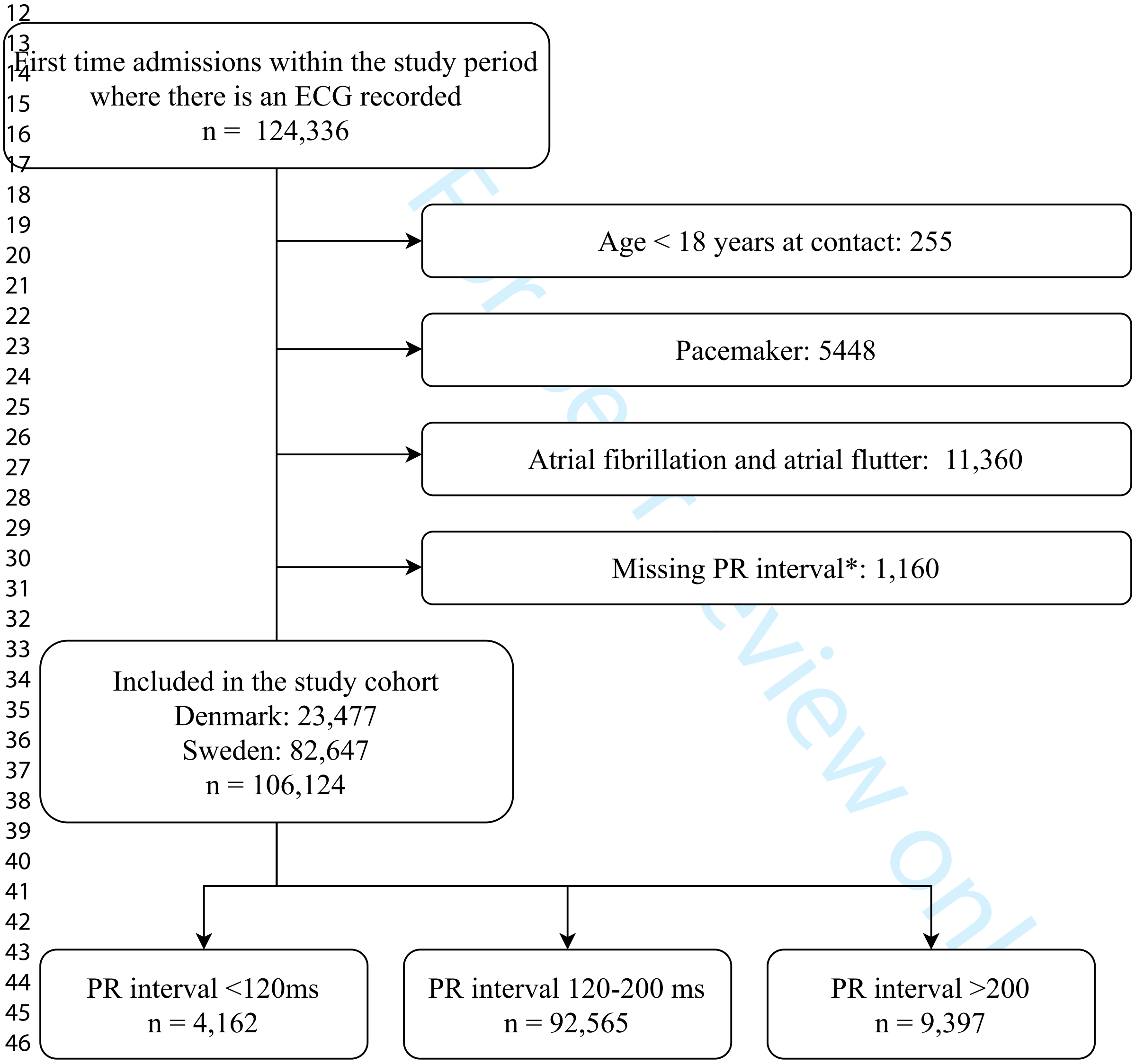
First time admissions within the study period
where there is an ECG recorded
n = 124,336
Age < 18 years at contact: 255
Pacemaker: 5448
Atrial fibrillation and atrial flutter: 11,360
Missing PR interval*: 1,160
Included in the study cohort
Denmark: 23,477
Sweden: 82,647
n = 106,124
PR interval <120ms
n = 4,162
PR interval 120-200 ms
n = 92,565
PR interval >200
n = 9,397

Figure 2: Cubic Spline

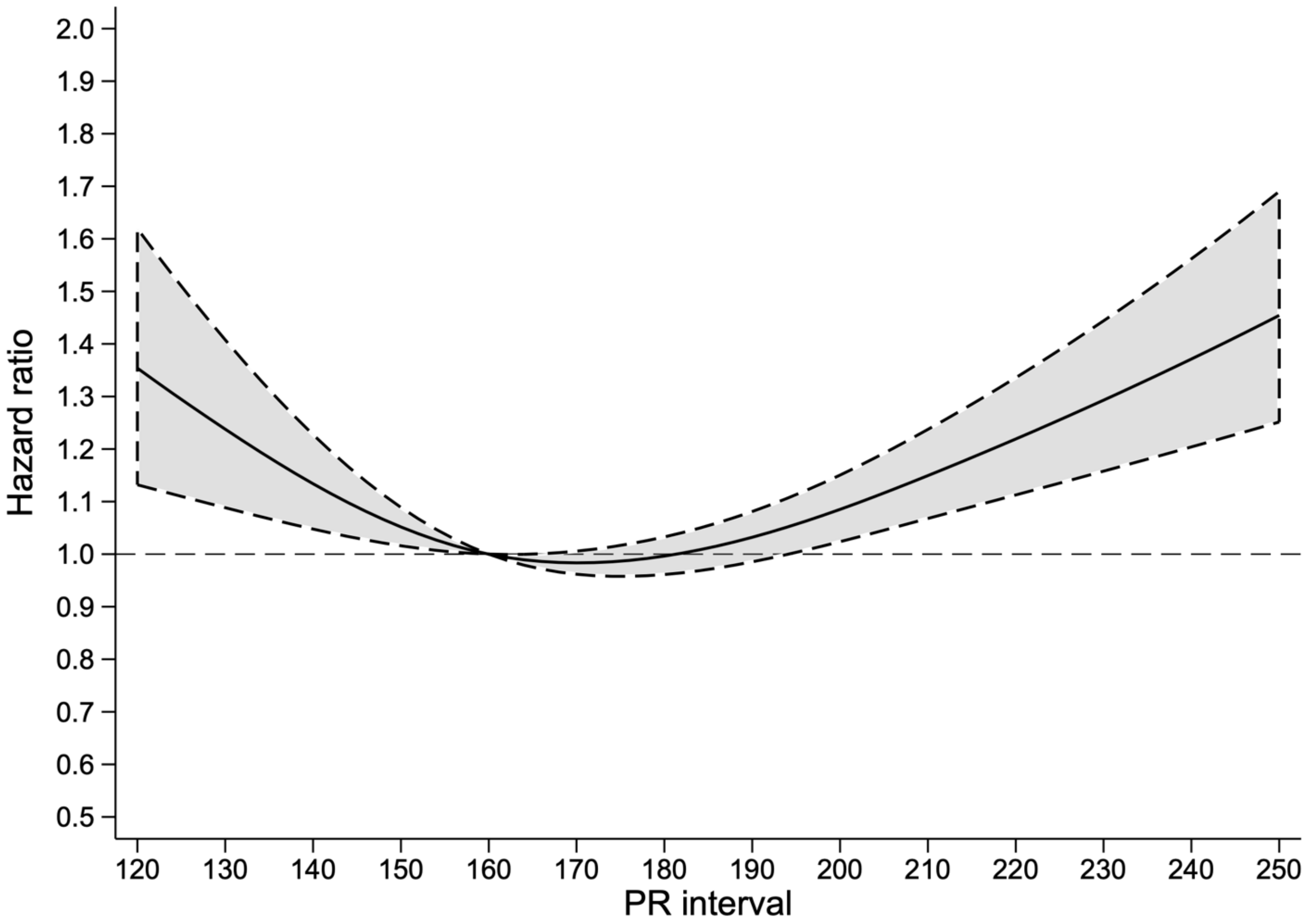

Figure 3: Kaplan-Meier

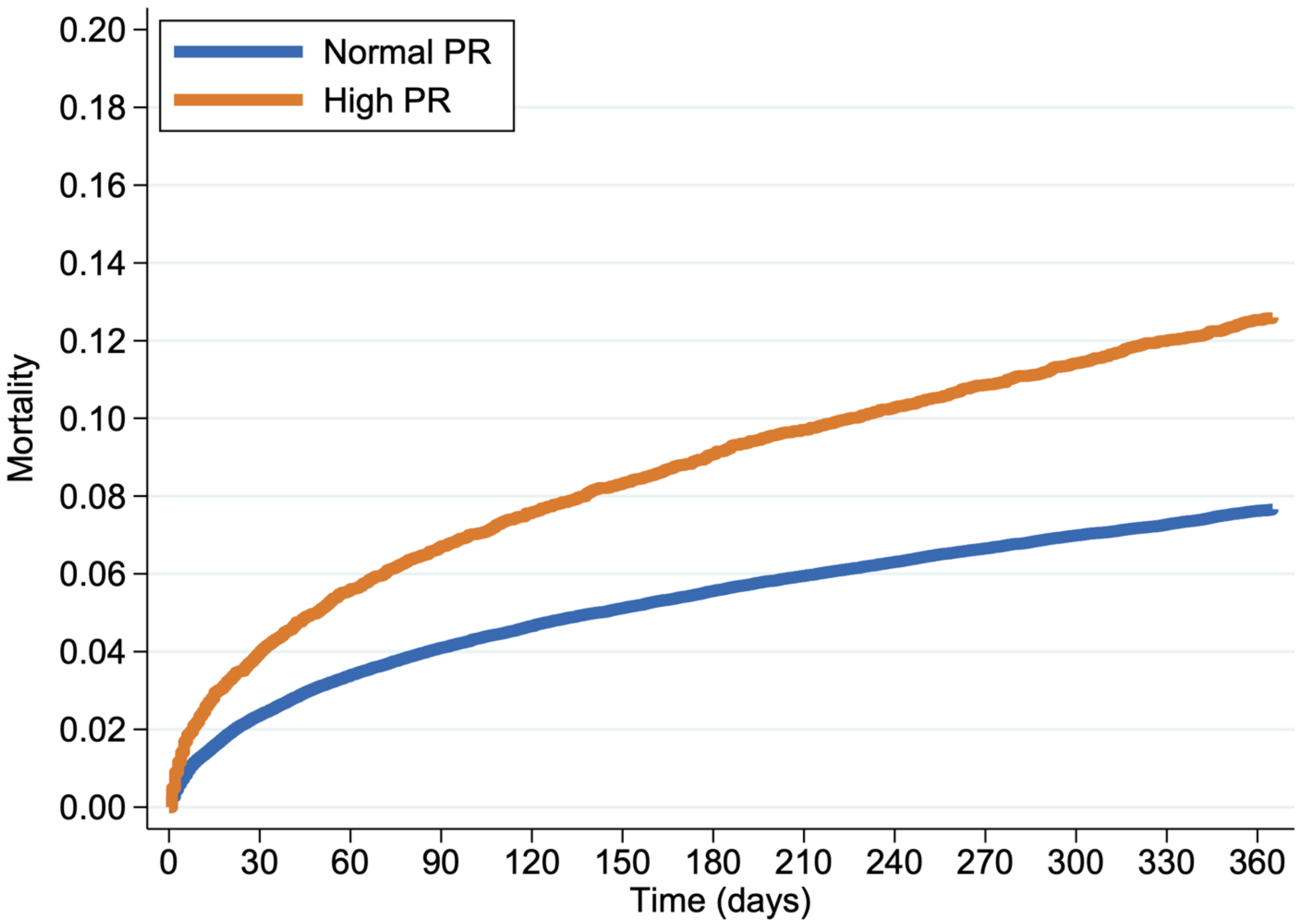

y only

Supplementary Appendix

PR interval prolongation and one-year mortality in the emergency department: a multicenter transnational cohort study

Supplementary materials and methods

Appendix A – Data sources

ECGs were extracted from MUSE® Cardiology Information System (GE Healthcare, Wauwatosa, WI, USA) or Megacare Electrocardiographic (ECG) Management System (Simens-Elema, Stockholm, Sweden). Information regarding time of arrival to the ED, triage, course of admission, length of stay, and time of discharge were extracted from the logistic systems in the ED at the Region of Southern Denmark [1] and Region of Skåne.

A unique ten-digit personal civil registration number given in Denmark and Sweden allows individual cross-linkage between databases. Data regarding birth, emigration, and vital status was extracted from the Danish Civil Registration System [2] and the Swedish Population Registry [3].

Data concerning patient comorbidities were retrieved from the Danish National Patient Registry [4] and the Skåne Healthcare Register [5] which contains diagnoses from both primary and secondary healthcare sectors. Data regarding prescriptions came from the Danish National Prescription Registry [4] and the Swedish National Pharmacy Register [6].

For peer review only

Appendix B – ECG measurements

Danish ECGs were recorded and digitally stored in MUSE® Cardiology Information System (GE Healthcare, Wauwatosa, WI, USA) and processed by the Marquette 12SL algorithm[7]. Swedish ECG's were stored by either Marquette or Philips Diagnostic ECG (Philips) and analyzed by the DXL algorithm [8]. ECGs were recorded by pre-hospital medical staff or at either one of the two clinics and all patients who had an ECG recorded within +/- 4 hours of admission to the ED were included. In the case of multiple ECGs recorded on the same individual, the first ECG of acceptable quality was used in the study. Individuals presenting with the following ECGs, obtained by the 12SL algorithm, were excluded from the study: pacemaker or Implantable Cardioverter-Defibrillator (ICD) who had not been excluded by previous criteria (n = 5,448), those with atrial fibrillation and atrial flutter (n = 11,360), and missing PR intervals (n = 1,160). Missing PR interval on the ECG was due to bad quality ECG or nodal rhythm on ECG. Pacemaker or ICD were defined as previous history of pacemaker or ICD implantation (Z95.0) and/or pacemaker rhythm recorded on the ECG.

Furthermore, patients presenting with a PR interval <120 ms (n = 4,162) were excluded from the analysis in order to evaluate PR interval prolongation compared to a normal PR interval of 120-200 ms. The Marquette 12SL algorithm calculates a median PR interval from the 10-second tracing ECG starting at the lead showing the earliest depolarization of the atria to the earliest depolarization of the ventricles in any lead [7]

The ability of the 12SL algorithm to determine PR interval has been evaluated by GE in the Physician's Guide [7] and by Nielsen et al[9], who found a mean difference between manual and 12SL measured PR interval of -0,21 ms (95% CI, -1.17 ms to 0.76 ms) with limits of agreement (±2 standard deviations) ranging from -14.1 ms to 13.7 ms. The ability of the Marquette 12SL algorithm to determine the presence of atrial fibrillation has been validated by Hansen et al [10].

The mean PR interval measured in the cohort were 161.0 ms (SD: 29,35). The GE Marquette 12SL measured a mean of 160,6 ms (SD: 29.27) and the Philips DXL a mean of 161,54 ms (SD: 29,45). Figure B1 shows a visual presentation of the PR intervals measured by the two different algorithms. As mention GE Marquette 12SL has been validated, and the above show a strong correlation between the two algorithms.

Figure B:

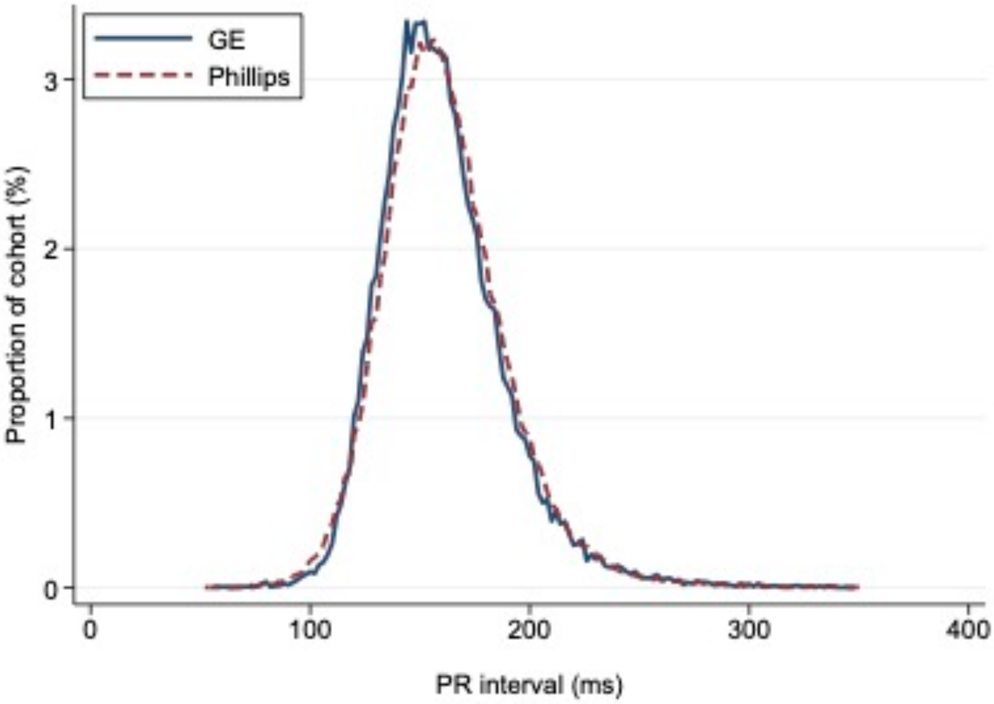

Distribution of the PR intervals measured by GE Marquette 12 SL (blue) and Philips DXL (red)

For peer review only

Appendix C – Codes and definitions

C.1: Charlson Comorbidity Index*

Condition	Assigned weight	ICD-10 codes
**Peripheral vascular disease**	1	I70.x, I71x, I73.1, I73.8, I73.9, I77.1, I79.0, I79.2, K55.1, K55.8, K55.9, Z95.8, Z95.9
**Cerebrovascular disease**	1	G45.x, G46.x, H34.0, I60.x-I69.x
**Dementia**	1	F00.x-F03.x, F05.1, G30.x, G31.1
**Chronic pulmonary disease**	1	I27.8, I27.9, J40.x-J47.x, J60.x-J67.x, J68.4, J70.1, J70.3
**Rheumatic disease**	1	M05.x, M06.x, M31.5, M32.x-M34.x, M35.1, M35.3, M36.0
**Peptic ulcer disease**	1	K25.x-K28.x
**Mild liver disease**	1	B18.x, K70.0-K70.3, K70.9, K71.3-K71.5, K71.7, K73.x, K74.x, K76.0, K76.2-K76.4, K76.8, K76.9, Z94.4
**Diabetes without chronic complications**	1	E10.0, E10.1, E10.6, E10.8, E10.9, E11.0, E11.1, E11.6, E11.8, E11.9, E12.0, E12.1, E12.6, E12.8, E12.9, E13.0, E13.1, E13.6, E13.8, E13.9, E14.0, E14.1, E14.6, E14.8, E14.9
**Hemiplegia or paraplegia**	2	G04.1, G11.4, G80.1, G80.2, G81.x, G82.x, G83.0-G83.4, G83.9
**Renal disease**	2	I12.0, I13.1, N03.2-N03.7, N05.2- N05.7, N18.x, N19.x, N25.0, Z49.0-Z49.2, Z94.0, Z99.2
**Diabetes with chronic complications**	2	E10.2-E10.5, E10.7, E11.2-E11.5, E11.7, E12.2-E12.5, E12.7, E13.2- E13.5, E13.7, E14.2-E14.5, E14.7
**Cancer**	2	C00.x-C26.x, C30.x-C34.x, C37.x- C41.x, C43.x, C45.x-C58.x, C60.x-C76.x, C81.x-C85.x, C88.x, C90.x-C97.x
**Metastatic cancer**	3	C77.x-C80.x
**Moderate or severe liver disease**	3	I85.0, I85.9, I86.4, I98.2, K70.4, K71.1, K72.1, K72.9, K76.5, K76.6, K76.7
**AIDS/HIV****	6	B20.x-B22.x, B24.x

**Diagnostic codes for myocardial infarction and heart failure are not included in the index but are included separately in this study as covariates.*
*** AIDS = acquired immunodeficiency syndrome, HIV = human immunodeficiency virus*

C.2: Myocardial infarction and congestive heart failure*

**Myocardial infarction**	I21.x, I22.x, I25.2
**Congestive heart failure**	I09.9, I11.0, I13.0, I13.2, I25.5, I42.0, I42.5-I42.9, I43.x, I50.x, P29.0

** Defined by Charlson Comorbidity Index but included separately in the study as covariates*

C.3: List of drugs associated with PR interval prolongation*

**Beta blocking agents**	C07*
**Calcium channel blockers**	C08*
**Digitalis glycosides**	C01AA*
**Adenosine**	C01EB10

**Usage of drugs are included dichotomously in the analysis defined as a redeemed prescription within 90 days of admission to the emergency department*

For peer review only

Appendix D – Supplementary results

Propensityscore matched cohort only adjusting for age and sex.

Tabel D.1,

	n	Events	HR (CI 95%)
Propensity score matched cohort*			
One-year all-cause mortality			
Normal PR 120-200 ms	24,996	3,248	1.0 (ref)
Prolonged PR >200 ms	8,321	893	0.96 (0.89-1.03)

For peer review only

Table D.2, sensitivity analysis

	Study cohort				Matched cohort	
	All	Prolonged PR >200 ms	Normal PR 120-180 ms	LowPR <120 ms	Prolonged PR >200 ms	Normal PR 120-180 ms
All	(n=92,089)	(n=9,397)	(n=78,530)	(n=4,162)	(n=7,016)	(n=21,048)
Male sex	43,648 (47.4%)	5,558 (59.1%)	36,521 (46.5%)	1,569 (37.7%)	3,621 (51.6%)	10,844 (51.5%)
Age, median (IQR)	60 (42-73)	75 (63-84)	58 (41-71)	52 (34-69)	70 (58-78)	69 (58-78)
18-50	33,693 (36.6%)	1,157 (12.3%)	30,537 (38.9%)	1,999 (48.0%)	1,157 (16.5%)	3,494 (16.6%)
51-69	29,430 (32.0%)	2,373 (25.3%)	25,886 (33.0%)	1,171 (28.1%)	2,349 (33.5%)	7,077 (33.6%)
70+	28,966 (31.5%)	5,867 (62.4%)	22,107 (28.2%)	992 (23.8%)	3,510 (50.0%)	10,477 (49.8%)
Charlson						
0	64,843 (70.4%)	5,785 (61.6%)	56,140 (71.5%)	2,918 (70.1%)	4,540 (64.7%)	13,584 (64.5%)
1	11,998 (13.0%)	1,309 (13.9%)	10,114 (12.9%)	575 (13.8%)	993 (14.2%)	2,983 (14.2%)
2	9,495 (10.3%)	1,494 (15.9%)	7,622 (9.7%)	379 (9.1%)	911 (13.0%)	2,804 (13.3%)
3+	5,753 (6.2%)	809 (8.6%)	4,654 (5.9%)	290 (7.0%)	572 (8.2%)	1,677 (8.0%)
Other diagnoses						
Heart failure	3,651 (4.0%)	950 (10.1%)	2,534 (3.2%)	167 (4.0%)	276 (3.9%)	839 (4.0%)
MI	4,019 (4.4%)	913 (9.7%)	2,967 (3.8%)	139 (3.3%)	351 (5.0%)	1,120 (5.3%)
Use of medication						
QT-prolong drugs	15,281 (16.6%)	2,886 (30.7%)	11,912 (15.2%)	483 (11.6%)	1,638 (23.3%)	4,961 (23.6%)
Center						
Odense	11,850 (12.9%)	1,146 (12.2%)	10,162 (12.9%)	542 (13.0%)	886 (12.6%)	2,658 (12.6%)
South West Jutland	8,559 (9.3%)	932 (9.9%)	7,337 (9.3%)	290 (7.0%)	693 (9.9%)	2,079 (9.9%)
Skåne	37,541 (40.8%)	3,960 (42.1%)	31,682 (40.3%)	1,899 (45.6%)	2,801 (39.9%)	8,403 (39.9%)
Helsingborg	34,139 (37.1%)	3,359 (35.7%)	29,349 (37.4%)	1,431 (34.4%)	2,636 (37.6%)	7,908 (37.6%)
ECG HR, median (IQR)	77 (66-90)	71 (62-83)	77 (67-90)	85 (70-103)	71 (61-83)	78 (67-91)

Supplementary results showing baseline characteristics in patients with normal PR interval (120-180 ms) and PR interval prolongation (>200 ms).

Appendix E – Post hoc analysis

Cardiovascular events

	n	Events	HR (CI 95%)
Propensity score matched cohort			
One year risk of MI			
Normal PR 120-200 ms	22,862	1,354	1.0 (ref)
Prolonged PR >200 ms	7,651	521	1.26 (1.07-1.49)
One year risk of CHF			
Normal PR 120-200 ms	23,084	1,238	1.0 (ref)
Prolonged PR >200 ms	7,724	456	1.16 (1.01-1.34)

For peer review only

Association between PR interval prolongation and myocardial infarction (MI) and congestive heart failure (CHF). MI and CHF are defined as listed under table C.2

References

1. Norgaard B, Mogensen CB, Teglbjaerg LS, et al. Diagnostic packages can be assigned accurately in emergency departments. A multi-centre cohort study. *Dan Med J* 2016;63(6) [published Online First: 2016/06/07]
2. Schmidt M, Pedersen L, Sorensen HT. The Danish Civil Registration System as a tool in epidemiology. *Eur J Epidemiol* 2014;29(8):541-9. doi: 10.1007/s10654-014-9930-3 [published Online First: 2014/06/27]
3. Ludvigsson JF, Otterblad-Olausson P, Pettersson BU, et al. The Swedish personal identity number: possibilities and pitfalls in healthcare and medical research. *Eur J Epidemiol* 2009;24(11):659-67. doi: 10.1007/s10654-009-9350-y [published Online First: 2009/06/09]
4. Schmidt M, Schmidt SA, Sandegaard JL, et al. The Danish National Patient Registry: a review of content, data quality, and research potential. *Clin Epidemiol* 2015;7:449-90. doi: 10.2147/clep.s91125 [published Online First: 2015/11/26]
5. Löfvendahl S, Schelin MEC, Jöud A. The value of the Skåne Health-care Register: Prospectively collected individual-level data for population-based studies. *Scand J Public Health* 2020;48(1):56-63. doi: 10.1177/1403494819868042 [published Online First: 2019/12/10]
6. Astrand B, Hovstadius B, Antonov K, et al. The Swedish National Pharmacy Register. *Stud Health Technol Inform* 2007;129(Pt 1):345-9. [published Online First: 2007/10/04]
7. Marquette 12SL ECG Analysis Program: Physician's Guide. Revision B 2056246-002. https://www.gehealthcare.com: GE Healthcare, 2015:428.
8. Philips DXL ECG Algorithm Physician's Guide. 2 ed. Andover MA: Philips Medical Systems, 2009.
9. Nielsen JB, Pietersen A, Graff C, et al. Risk of atrial fibrillation as a function of the electrocardiographic PR interval: results from the Copenhagen ECG Study. *Heart Rhythm* 2013;10(9):1249-56. doi: 10.1016/j.hrthm.2013.04.012 [published Online First: 2013/04/24]
10. Hansen TG, AP MS, Brandes A, et al. New-onset atrial fibrillation among patients with infection in the emergency department: A multicentre cohort study of one-year stroke risk. *Am J Med* 2019 doi: 10.1016/j.amjmed.2019.06.048 [published Online First: 2019/08/14]

For peer review only

STROBE Statement—Checklist of items that should be included in reports of ***cohort studies***

	Item No	Recommendation	Page No
**Title and abstract**	1	(*a*) Indicate the study's design with a commonly used term in the title or the abstract<br>(*b*) Provide in the abstract an informative and balanced summary of what was done and what was found	1-3
**Introduction**			
Background/rationale	2	Explain the scientific background and rationale for the investigation being reported	4
Objectives	3	State specific objectives, including any prespecified hypotheses	4
**Methods**			
Study design	4	Present key elements of study design early in the paper	5
Setting	5	Describe the setting, locations, and relevant dates, including periods of recruitment, exposure, follow-up, and data collection	5
Participants	6	(*a*) Give the eligibility criteria, and the sources and methods of selection of participants. Describe methods of follow-up<br>(*b*) For matched studies, give matching criteria and number of exposed and unexposed	5
Variables	7	Clearly define all outcomes, exposures, predictors, potential confounders, and effect modifiers. Give diagnostic criteria, if applicable	5-6
Data sources/ measurement	8*	For each variable of interest, give sources of data and details of methods of assessment (measurement). Describe comparability of assessment methods if there is more than one group	5-6
Bias	9	Describe any efforts to address potential sources of bias	6
Study size	10	Explain how the study size was arrived at	5-6
Quantitative variables	11	Explain how quantitative variables were handled in the analyses. If applicable, describe which groupings were chosen and why	6
Statistical methods	12	(*a*) Describe all statistical methods, including those used to control for confounding<br>(*b*) Describe any methods used to examine subgroups and interactions<br>(*c*) Explain how missing data were addressed<br>(*d*) If applicable, explain how loss to follow-up was addressed<br>(*e*) Describe any sensitivity analyses	6-7
**Results**			
Participants	13*	(a) Report numbers of individuals at each stage of study—eg numbers potentially eligible, examined for eligibility, confirmed eligible, included in the study, completing follow-up, and analysed<br>(b) Give reasons for non-participation at each stage<br>(c) Consider use of a flow diagram	8
Descriptive data	14*	(a) Give characteristics of study participants (eg demographic, clinical, social) and information on exposures and potential confounders<br>(b) Indicate number of participants with missing data for each variable of interest<br>(c) Summarise follow-up time (eg, average and total amount)	8
Outcome data	15*	Report numbers of outcome events or summary measures over time	8

For peer review only

For peer review only - http://bmjopen.bmj.com/site/about/guidelines.xhtml

Main results	16	(*a*) Give unadjusted estimates and, if applicable, confounder-adjusted estimates and their precision (eg, 95% confidence interval). Make clear which confounders were adjusted for and why they were included<br>(*b*) Report category boundaries when continuous variables were categorized<br>(*c*) If relevant, consider translating estimates of relative risk into absolute risk for a meaningful time period	8
Other analyses	17	Report other analyses done—eg analyses of subgroups and interactions, and sensitivity analyses	8
**Discussion**			
Key results	18	Summarise key results with reference to study objectives	8-9
Limitations	19	Discuss limitations of the study, taking into account sources of potential bias or imprecision. Discuss both direction and magnitude of any potential bias	9-10
Interpretation	20	Give a cautious overall interpretation of results considering objectives, limitations, multiplicity of analyses, results from similar studies, and other relevant evidence	9-10
Generalisability	21	Discuss the generalisability (external validity) of the study results	9-10
**Other information**			
Funding	22	Give the source of funding and the role of the funders for the present study and, if applicable, for the original study on which the present article is based	1

*Give information separately for exposed and unexposed groups.

Note: An Explanation and Elaboration article discusses each checklist item and gives methodological background and published examples of transparent reporting. The STROBE checklist is best used in conjunction with this article (freely available on the Web sites of PLoS Medicine at http://www.plosmedicine.org/, Annals of Internal Medicine at http://www.annals.org/, and Epidemiology at http://www.epidem.com/). Information on the STROBE Initiative is available at http://www.strobe-statement.org.

For peer review only

For peer review only - http://bmjopen.bmj.com/site/about/guidelines.xhtml
